# Sustainable synthesis of Ag/UiO-66 composites using recycled silver for efficient adsorption of methylene blue and crystal violet

**DOI:** 10.1038/s41598-026-65189-x

**Published:** 2026-08-11

**Authors:** Hazem Ibrahem, Hassanien Gomaa, Ahmed A. Gahlan, Adham M. Nagiub

**Affiliations:** Department of Chemistry, Faculty of Science, Al-Azhar University, Assiut, 71524 Egypt

**Keywords:** Ag/UiO-66, Metal–organic framework, Recycled silver, Cationic dye adsorption, Methylene blue, Crystal violet, Chemistry, Environmental sciences, Materials science

## Abstract

Synthetic dyes are persistent and toxic water pollutants that require efficient removal technologies, and metal–organic frameworks (MOFs) have emerged as promising adsorbents for this purpose. In this work, a defect-engineered zirconium-based UiO-66 framework was synthesized by an HCl-modulated solvothermal route. UiO-66 was then functionalized with silver nanoparticles through a post-synthetic impregnation–reduction method to form an Ag/UiO-66 composite for the removal of the cationic dyes methylene blue (MB) and crystal violet (CV). The silver was recovered from electrical contact waste, thereby adding a resource-recovery dimension to the synthesis. Powder X-ray diffraction, XPS, FTIR, SEM, and TEM supported the incorporation of metallic Ag⁰ nanoparticles (about 3.3 nm; 3.11 wt%), uniformly dispersed on the crystals, with full preservation of the UiO-66 framework. Silver loading decreased the BET surface area from 870 to 642 m^2^ g^−1^, indicating partial pore occupation. Nevertheless, Ag/UiO-66 exhibited higher Langmuir capacities than pristine UiO-66, reaching 113.49 and 46.72 mg g^−1^ for MB and CV, respectively, compared with 104.98 and 43.72 mg g^−1^ for the pristine framework. Removal efficiencies above 98% were achieved at neutral pH, with equilibrium attained within 45 min. The adsorption was favored above the point of zero charge (pH_pzc_ = 5.88) through electrostatic attraction, followed the pseudo-second-order kinetic model, and was best described by the Langmuir isotherm (R^2^ > 0.98), indicating monolayer adsorption. Thermodynamic analysis indicated a spontaneous, exothermic process, with ΔH° values ranging from − 4.91 to − 9.31 kJ mol^−1^. The combined isotherm, kinetic, and spectroscopic results indicated that the uptake occurs through both physical and chemical interactions, including electrostatic attraction, π–π stacking, hydrogen bonding, and binding at the silver and defect sites. The composite retained over 90% removal after five adsorption–desorption cycles and removed 95.3% of MB and 93.6% of CV from Nile River water. These results demonstrate that silver recovered from electronic waste can be used to prepare effective, reusable Ag/UiO-66 adsorbents for the removal of cationic dyes.

**Supplementary Information:**

The online version contains supplementary material available at 10.1038/s41598-026-65189-x.

## Introduction

Water resources are increasingly threatened by contamination arising from rapid industrialization, urban development, and intensive agriculture, which accelerate the discharge of pollutants into freshwater systems^1–4^. Industrial effluents and agricultural runoff are major sources of this pollution, introducing heavy metals, pharmaceuticals, pesticides, and synthetic dyes that pose long-term risks to ecosystems and human health^5–7^. Among these pollutants, synthetic dyes are of particular concern because of their complex molecular structures, high chemical stability, and resistance to biodegradation^8,9^. Today, dyes are indispensable in industries such as textiles, printing, plastics, cosmetics, food processing, and medicine, with global production estimated at 0.7–1.0 million tons per year and more than 100,000 commercial dyes available^10,11^. This widespread utilization has resulted in toxic and persistent compounds being released into aquatic environments, significantly harming water quality^12,13^. Even at low concentrations, dyes impart intense color, reduce light penetration, and disrupt photosynthesis in aquatic ecosystems^14,15^.

According to their ionic nature, dyes are commonly classified as cationic, anionic, or non-ionic^16,17^. Among these, cationic dyes such as methylene blue (MB) and crystal violet (CV) are widely used because of their strong affinity for negatively charged substrates^18,19^. However, their persistence, aquatic toxicity, and ability to induce oxidative stress and genotoxic effects have raised serious environmental concerns^20–22^. The development of efficient methods for their removal from wastewater is therefore of considerable importance. Conventional dye treatment technologies are generally grouped into chemical, biological, and physical methods. Chemical methods rely on oxidative degradation processes such as ozonation, Fenton reactions, photochemical oxidation, and electrochemical treatment, whereas biological methods use microbial or enzymatic degradation^23–25^. Physical methods, including membrane filtration and adsorption, remove dyes without changing their molecular structure. Among these, adsorption is widely regarded as one of the most effective and environmentally friendly techniques, owing to its simplicity, fast kinetics, and absence of harmful by-products^26,27^.

Metal–organic frameworks (MOFs) have attracted increasing attention as advanced adsorbents due to their high surface areas, tunable porosity, and versatile surface chemistry^28,29^. These porous crystalline materials, built from metal nodes and organic linkers, have been widely explored for catalysis, gas storage, dye removal, sensing, and energy-related applications^30–33^. In particular, the zirconium-based UiO-66 framework is distinguished by its exceptional hydrothermal and chemical stability, which arises from strong Zr–O coordination bonds^34–36^. Its high surface area, structural robustness, and capacity for defect engineering make UiO-66 a promising adsorbent for water treatment^37–39^. The surface chemistry of MOFs can be tuned in two main ways. The first is pre-synthetic modification, in which functionalized linkers or mixed-metal systems are used to directly incorporate the desired function into the framework. During synthesis, modulators are added to control crystal nucleation, growth, and defect formation. The second is post-synthetic modification, which is carried out after the MOF has formed and allows surface reactions, decoration with additional species, metalation, or encapsulation of guest molecules. These strategies allow MOFs to be tailored with a wide range of molecules, metals, or functional groups to improve their performance in adsorption and other applications^40–42^.

Several studies have shown that incorporating metal nanoparticles, including silver, can enhance the functionality of UiO-66. Yang and co-workers anchored Cu(II) species onto the defective –OH/–OH_2_ sites of UiO-66 and reduced them under H_2_ to obtain Cu/UiO-66 with enhanced NO_2_ reduction activity^43^. Sun and colleagues used a post-synthetic exchange strategy to prepare Ag/Cu-BTC for the oxidation of toluene to benzaldehyde, where the performance depended on the partial substitution of Ag for Cu^44^. Li and co-workers deposited Ag nanoparticles onto UiO-66 by NaBH_4_ reduction and reported improved catalytic activity in styrene oxidation^45^. Beyond catalysis, Salama et al. prepared Ag/UiO-66 by an impregnation–reduction method and applied it to the adsorption of anionic dyes^46^, while Chiericatti and co-workers reported strong antifungal activity for Ag/UiO-66 prepared by simple impregnation^47^. Silver is therefore a valuable additive for UiO-66 in water treatment due to its antibacterial properties and functional role in adsorption. Metallic Ag⁰ nanoparticles do not themselves act as high-capacity dye adsorbents. Their primary benefit is not derived from additional surface area—which typically decreases upon nanoparticle loading—but from the generation of new active sites at the Ag⁰/framework interface. These interfacial sites facilitate π–π, n–π, and coordinative interactions with dye molecules^46^. In synergy with the terminal –OH groups and coordinatively unsaturated Zr sites exposed by HCl-induced defects, these features enhance the framework’s affinity for cationic dyes, even as the overall surface area declines^46^.

In this study, Ag/UiO-66 composites were prepared using silver recovered from waste electrical contact straps and evaluated as efficient, reusable adsorbents for the removal of MB and CV as model cationic pollutants. The recovery and reuse of metals in functional materials is a well-established strategy; the contribution of this work therefore lies not in the concept of recycling itself, but in an integrated route that converts silver reclaimed from end-of-life electrical contacts into a functional AgNO_3_ precursor, incorporates it into UiO-66, and applies the resulting composite to dye removal. Although Ag-loaded UiO-66 materials have been reported, the adsorption of MB and CV on Ag-loaded, defect-modulated UiO-66 under dark conditions has, to our knowledge, received little attention. A further point of interest is that metallic Ag⁰ nanoparticles are not intrinsically high-capacity adsorbents, yet the composite adsorbs more dye than pristine UiO-66 despite its lower surface area; this suggests that uptake is influenced by active sites at the Ag⁰/framework interface and at HCl-generated defects rather than by surface area alone. To explore these points, HCl-modulated UiO-66 was combined with nanoparticles prepared from recovered silver, and the resulting composite was characterized and evaluated through adsorption kinetics, isotherm, thermodynamic, and reusability studies. This work provides new insight into the adsorption of cationic dyes on Ag/UiO-66 composites and offers a more sustainable route for preparing Ag-modified UiO-66 adsorbents.

## Experimental

### Materials

All chemicals were of analytical grade and were used as received. Dimethylformamide (DMF), MB, and CV were purchased from Alpha Chemika. Terephthalic acid (1,4-benzenedicarboxylic acid, C_8_H_6_O_4_), anhydrous zirconium chloride (ZrCl_4_), and sodium borohydride (NaBH_4_) were purchased from Sigma-Aldrich. Copper bars were purchased from Goodfellow Advanced Materials. Sodium hydroxide (NaOH), hydrochloric acid (HCl), and absolute ethanol (C_2_H_5_OH) were purchased from Merck. Distilled water was used throughout the experiments.

### Preparation of the silver precursor

Silver-bearing contact materials were separated manually from an ABB SACE Tmax T3N 250 molded-case circuit breaker (MCCB) and associated relays (Fig. S1). These contacts are typically manufactured from silver-based contact alloys rather than pure silver, although the exact alloy composition of the recovered contacts was not determined in the present study. The straps were dissolved in a mixture of distilled water (70 mL) and concentrated nitric acid (68%; 30 mL) at 60–85 °C. The solution was then filtered to remove insoluble residues originating from the non-silver constituents of the contact material. Metallic silver was recovered by cementation, in which copper bars were immersed in the filtrate for 24 h. Because silver has a higher standard reduction potential than copper (Cu + 2Ag^+^ → Cu^2+^ + 2Ag), Ag^+^ ions are selectively reduced to metallic silver, whereas less noble metal ions largely remain in solution. The silver was washed thoroughly with hot water, dried, and ground into a fine powder. To prepare the precursor, about 0.64 g of the recovered silver was dissolved in dilute nitric acid (1:1 v/v HNO_3_:H_2_O) with gentle heating, yielding 1 g of AgNO_3_ (Scheme 1.1). The solution was evaporated to dryness, and the AgNO_3_ crystals were stored in a dark desiccator. The crystals were subsequently dissolved in distilled water and diluted to 128 mL to give a 0.046 M AgNO_3_ stock solution, of which a 15 mL aliquot was used for the synthesis of the Ag/UiO-66 composite^48,49^.

### Synthesis of UiO-66

UiO-66 was prepared following a reported method with minor modifications^50^. ZrCl_4_ (0.373 g, 1.60 mmol) and terephthalic acid (0.531 g, 3.20 mmol) were dissolved separately in DMF, using 50 mL for each precursor. Deionized water (0.5 mL) and hydrochloric acid (0.3 mL, 2.25 equiv. relative to Zr) were added to the ZrCl_4_ solution to promote defect formation. Both solutions were stirred for 30 min at room temperature and then combined. The mixture was transferred to a Teflon-lined autoclave and heated at 130 °C for 24 h. The white precipitate was collected by centrifugation, washed three times each with DMF and ethanol, and dried at 80 °C (Scheme 1.2).

### Synthesis of Ag/UiO-66 composite

UiO-66 (1.5 g) was dispersed in absolute ethanol (20 mL) and stirred in the dark for 15 min. An ethanolic AgNO₃ solution (15 mL, 0.046 M) was then added, and the suspension was stirred in the dark at room temperature for 2 h to load Ag^+^ ions onto the framework. The solid was separated by centrifugation, and the supernatant was decanted. The solid was redispersed in fresh cold ethanol (10 mL) and cooled in an ice bath (0–4 °C) for 15 min. A freshly prepared ice-cold NaBH_4_ solution in ethanol (13.9 mL, 200 mM) was then added dropwise over 30 min under vigorous stirring, giving a BH_4_^−^:Ag molar ratio of 4. The mixture was stirred for a further 30 min in the ice bath, then for 30 min at room temperature. The Ag/UiO-66 product was collected by centrifugation, washed three times with cold ethanol (10 mL each), and dried at 80 °C for 12 h (Scheme 1.3).

**Scheme 1:**
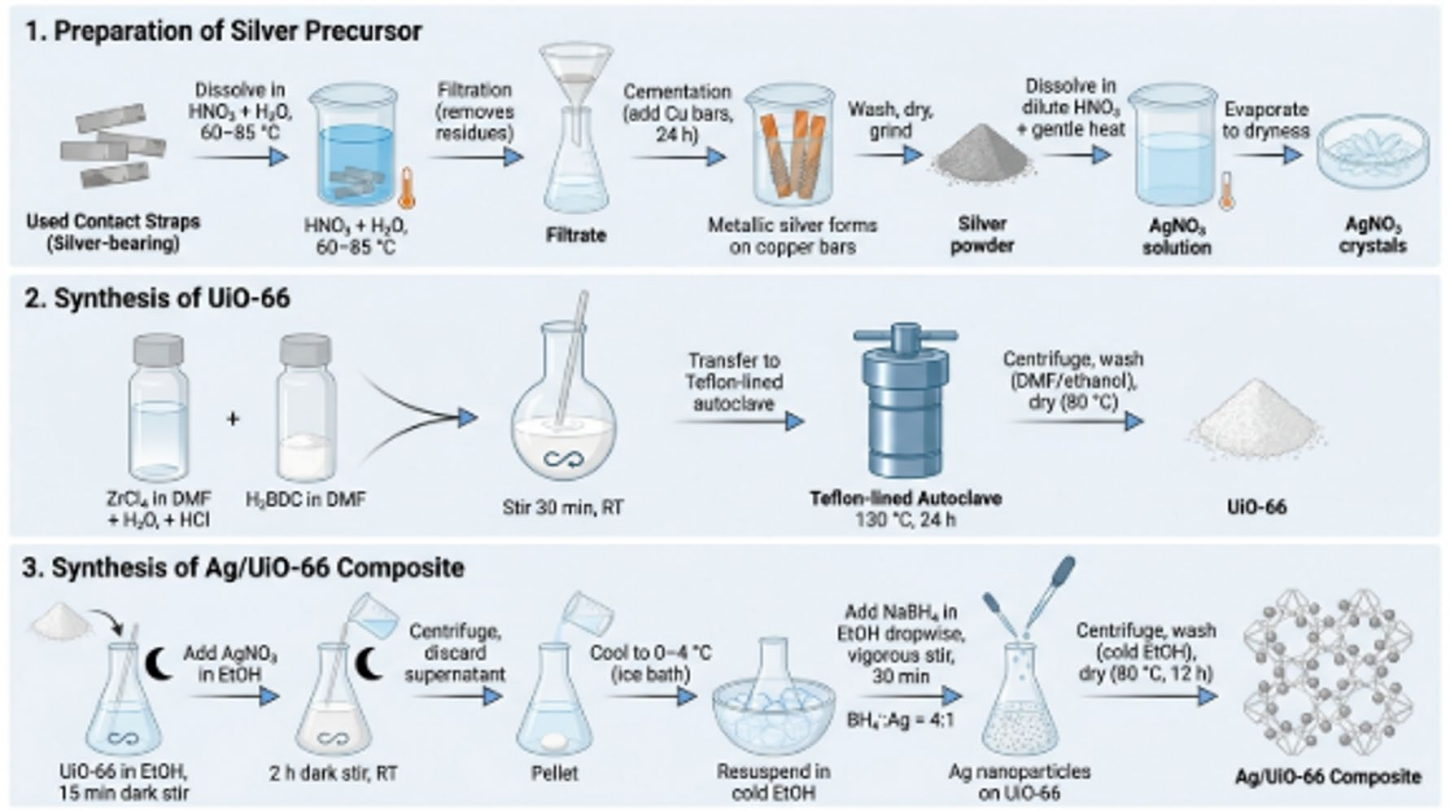
Preparation of the Ag/UiO-66 composite: (1) recovery of the AgNO_3_ precursor from used silver-bearing contact straps, (2) solvothermal synthesis of HCl-modulated UiO-66, and (3) AgNO_3_ impregnation and NaBH_4_ reduction to deposit Ag nanoparticles on UiO-66.

### Characterization tools

Powder X-ray diffraction (PXRD) patterns were collected to determine the crystalline structure of samples using a Philips PW1700 diffractometer (Eindhoven, Netherlands) with monochromated Cu Kα radiation (λ = 1.5418 Å), operated at 40 kV and 40 mA. The porosity and specific surface area of materials were characterized by nitrogen adsorption–desorption isotherms measured at 77.35 K using a NOVAtouch 2LX surface area and porosity analyzer (Quantachrome Instruments, USA). The morphology of the samples was examined by scanning electron microscopy (SEM) coupled with energy-dispersive X-ray spectroscopy (EDX) using a Sigma 500 VP microscope (Carl Zeiss, Germany). Transmission electron microscopy (TEM) images were obtained using a JEOL JEM-100CXII transmission electron microscope (JEOL, Japan). X-ray photoelectron spectroscopy (XPS) was collected on K-ALPHA (Thermo Fisher Scientific, USA) with monochromatic X-ray Al K-alpha radiation − 10 to 1350 eV, spot size 400 μm, at a pressure of 10^− 9^ mbar, with a full-spectrum pass energy of 200 eV and a narrow-spectrum 50 eV. Fourier transform infrared (FTIR) spectra were recorded using the KBr pellet technique over the wavenumber range of 400–4000 cm^−1^ with an Alpha FTIR spectrometer (Bruker, Germany). The surface charge of the Ag/UiO-66 composite was determined over the pH range of 2–11 using a Zetasizer Nano ZS (Model ZEN3600, Malvern Instruments, UK). The thermal stability of samples was evaluated by thermogravimetric analysis (TGA) using a DTG-60 H simultaneous DTA–TG analyzer (Shimadzu, Japan) under a nitrogen atmosphere with a gas flow rate of 40 mL min^−1^ and a heating rate of 10 °C min^−1^. The silver content was determined by inductively coupled plasma optical emission spectroscopy (ICP–OES) using Thermo Scientific iCAP 6300- (Thermo Fisher Scientific, USA). The dye absorbance was measured by a UV–Visible spectrophotometer (DR 6000, HACH, Germany).

### Batch adsorption studies

Calibration curves for MB and CV were prepared before the adsorption experiments. Stock solutions were prepared by dissolving 0.25 g of each dye in 250 mL of distilled water. Standard solutions of 10, 20, 30, 40, and 50 mg L^−1^ were then obtained by diluting the stock solutions. The absorbance of the blank and standard solutions was measured with a UV–Vis spectrophotometer from 200 to 700 nm. The maximum absorption wavelengths (λ_max_) were 658 nm for MB and 580 nm for CV. The calibration curves were obtained by plotting absorbance against dye concentration.

The adsorption experiments were carried out in 100 mL beakers under continuous stirring at 300 rpm. All experiments were performed in the dark at 25 °C, so that dye removal by modulated UiO-66 and Ag/UiO-66 occurred mainly through adsorption rather than photodegradation. For the pH study, 50 mg of adsorbent was added to 25 mL of MB or CV solution (10 mg L^−1^), and the pH was adjusted from 2.0 to 10.0 using small volumes of 1 mol L^−1^ HCl or NaOH. For the kinetic study, 50 mg of adsorbent was added to 25 mL of dye solution (10 mg L^−1^), and samples were taken at set time intervals (1–90 min). For the isotherm study, 50 mg of adsorbent was mixed with 25 mL of dye solution at concentrations from 10 to 300 mg L^−1^. The effect of adsorbent dose was studied by changing the adsorbent mass from 10 to 60 mg per 25 mL of solution. The effect of temperature was studied from 20 to 65 °C. After adsorption, the suspensions were centrifuged at 6000 rpm to separate the solid. The residual dye concentration was measured by UV–Vis spectroscopy at 658 nm for MB and 580 nm for CV. The removal efficiency (R%) was calculated using Eq. (1)^51^:

1 $$\:\begin{array}{c}R\%=\frac{100\left({\mathrm{C}}_{0}-{\mathrm{C}}_{\mathrm{e}}\right)}{{\mathrm{C}}_{0}} \end{array}$$

The equilibrium adsorption capacity (q_e_, mg g^−1^) was calculated using Eq. (2):

2 $$\:\begin{array}{c}{\mathrm{q}}_{\mathrm{e}}=\frac{\left({\mathrm{C}}_{0}-{\mathrm{C}}_{\mathrm{e}}\right)\mathrm{V}}{\mathrm{m}} \end{array}$$

where C_o_ and C_e_ are the initial and equilibrium dye concentrations (mg L^−1^), respectively, V is the solution volume (L), and m is the mass of adsorbent (g). All adsorption experiments were performed in triplicate, and the corresponding error bars are shown in the relevant figures.

## Results and discussion

### Physicochemical characterization of Ag/UiO-66

**Fig. 1:**
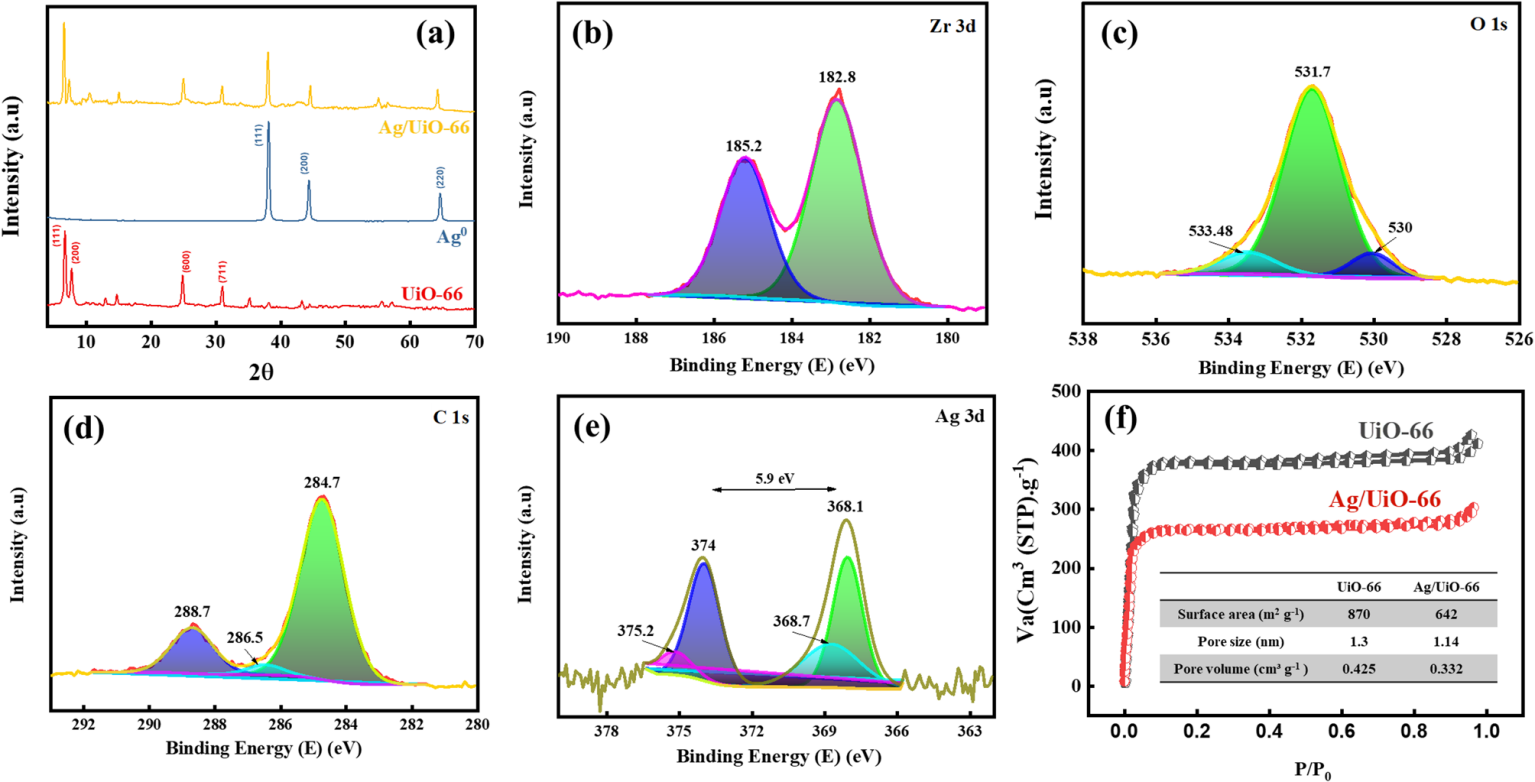
Structural and textural characterization of the prepared materials: (**a**) PXRD patterns of UiO-66, metallic silver (Ag⁰), and Ag/UiO-66; (**b**–**e**) high-resolution XPS spectra of Ag/UiO-66 showing the (**b**) Zr 3d, (**c**) O 1s, (**d**) C 1s, and (**e**) Ag 3d regions; and (**f**) N_2_ adsorption–desorption isotherms of UiO-66 and Ag/UiO-66, with the inset table listing the BET surface area, pore size, and pore volume.

Figure 1a shows the PXRD patterns of UiO-66, metallic Ag⁰, and the Ag/UiO-66 composite. UiO-66 showed sharp, well-defined reflections at 2θ ≈ 7.4°, 8.5°, 25.0°, and 30.9°, which are assigned to the (111), (200), (600), and (822) planes of its cubic framework. The sharpness and high intensity of these peaks indicate a well-crystallized material with long-range order. The pattern agrees with the reported PXRD pattern of UiO-66^52^ and confirms that the target framework was successfully formed. The metallic Ag⁰ pattern showed three reflections at 2θ ≈ 38.8°, 44.5°, and 64.7°, which are indexed to the (111), (200), and (220) planes of face-centered cubic silver (JCPDS No. 04-0783)^53,54^. These sharp Ag⁰ peaks confirm that the recovered silver was obtained as a crystalline, metallic phase. For the Ag/UiO-66 composite, the main framework peaks of UiO-66 were retained at 2θ ≈ 7.3°, 8.4°, 25.0°, and 30.9°, together with the characteristic Ag⁰ peaks at 2θ ≈ 38.0°, 44.6°, and 64.2°. The positions and relative intensities of the UiO-66 peaks remained nearly unchanged after Ag loading, indicating that the framework retained its crystallinity and was not damaged during impregnation and reduction. The Ag⁰ peaks in the composite were weaker and slightly broader than those of the bulk metallic silver. This is consistent with a low silver loading and with small, well-dispersed Ag nanoparticles. The presence of both sets of peaks indicates the successful formation of the Ag/UiO-66 composite, in which metallic Ag⁰ nanoparticles are supported on an intact UiO-66 framework.

XPS was used to study the elemental chemical states in Ag/UiO-66 and to assess whether the framework was preserved. The survey spectrum (Fig. S2) showed the O 1s, Zr 3p, Ag 3d, C 1s, and Zr 3d core-level peaks of the composite, confirming the presence of all expected elements. The Zr 3d spectrum (Fig. 1b) showed two peaks at 182.8 eV (Zr 3d_5_/_2_) and 185.2 eV (Zr 3d_3_/_2_), with a spin-orbit splitting of about 2.4 eV. Both peaks are assigned to Zr^4+^ in the Zr–O bonds of the Zr_6_ clusters. The positions and shape of the doublet match those of pristine UiO-66, indicating that the metal nodes were not reduced or altered during Ag loading and that the framework remained intact. The O 1s spectrum (Fig. 1c) was fitted with three components. The main peak at 531.7 eV is assigned to the Zr–O bonds of the framework. The component near 530 eV is assigned to lattice oxygen in the Zr–O–Zr nodes, and the shoulder at 533.48 eV is assigned to surface hydroxyl groups and adsorbed oxygen species such as water. The presence of these different oxygen environments is consistent with the defect-rich structure formed during HCl modulation, in which missing-linker sites expose extra hydroxyl and terminal oxygen groups. The C 1s spectrum (Fig. 1d) showed three components: aromatic C–C/C=C at 284.7 eV, C–O at 286.5 eV, and carboxylate O–C=O at 288.7 eV^55^. These three peaks match the carbon environments expected for the terephthalate linker. This shows that the organic linker kept its chemical structure after silver loading and that the reduction step did not damage the framework. The Ag 3d spectrum (Fig. 1e) showed a clear doublet at 368.1 eV (Ag 3d_5_/_2_) and 374.0 eV (Ag 3d_3_/_2_), with a spin-orbit splitting of 5.9 eV. This splitting is the characteristic value for metallic silver (Ag⁰) and agrees with reported values^56,57^. The absence of peaks that could be assigned to Ag^+^ or silver oxide indicates that the silver was fully reduced to the metallic state by the NaBH_4_ treatment. Taken together, the XPS results confirm that the silver in the composite is present as metallic Ag⁰ and that the UiO-66 framework was preserved after Ag loading.

The textural properties of modulated UiO-66 and Ag/UiO-66 were studied by N_2_ adsorption–desorption measurements at 77 K (Fig. 1f). Both samples showed type I(a) isotherms, with steep N_2_ uptake at low relative pressure (P/P_o_ < 0.05) that confirms their microporous structure^58^. A small hysteresis loop near P/P_o_ = 1 suggests a minor contribution from textural mesoporosity associated with interparticle voids rather than from framework mesopores, so that both materials remain intrinsically microporous. The modulated UiO-66 had a Brunauer–Emmett–Teller (BET) surface area of 870 m^2^ g^−1^ and a total pore volume of 0.425 cm^3^ g^−1^. After silver loading and reduction, the BET surface area of Ag/UiO-66 decreased to 642 m^2^ g^−1^, and the pore volume decreased to 0.332 cm^3^ g^−1^. The pore-size distributions obtained from the Barrett–Joyner–Halenda (BJH) method (Fig. S3) showed a main peak at about 1.3 nm for UiO-66 and about 1.1 nm for Ag/UiO-66, with smaller features at larger pore sizes. The reduction in surface area and pore volume suggests a partial loss of pore accessibility after Ag incorporation, possibly due to deposition near pore entrances, at defect regions, or on external crystal surfaces. A reduction in the apparent contribution of the larger defect-related pores is consistent with Ag deposition preferentially at these more accessible sites, which would also shift the measured pore-size distribution toward smaller values. This behavior is consistent with the association of Ag nanoparticles with the defect regions and external surfaces of UiO-66^59^. Although Ag loading reduced the surface area and pore volume, Ag/UiO-66 retained its microporous structure and still exhibited higher adsorption capacities for MB and CV than UiO-66. This shows that the higher dye uptake was not controlled by surface area alone, but by other factors, such as the new active sites introduced by the silver and the defect sites generated during HCl modulation, which may increase the framework’s affinity for the dyes. This point is supported quantitatively by the surface-area-normalized capacities discussed in “Adsorption mechanism”, which increase markedly after Ag loading even though the total surface area decreases.

**Fig. 2:**
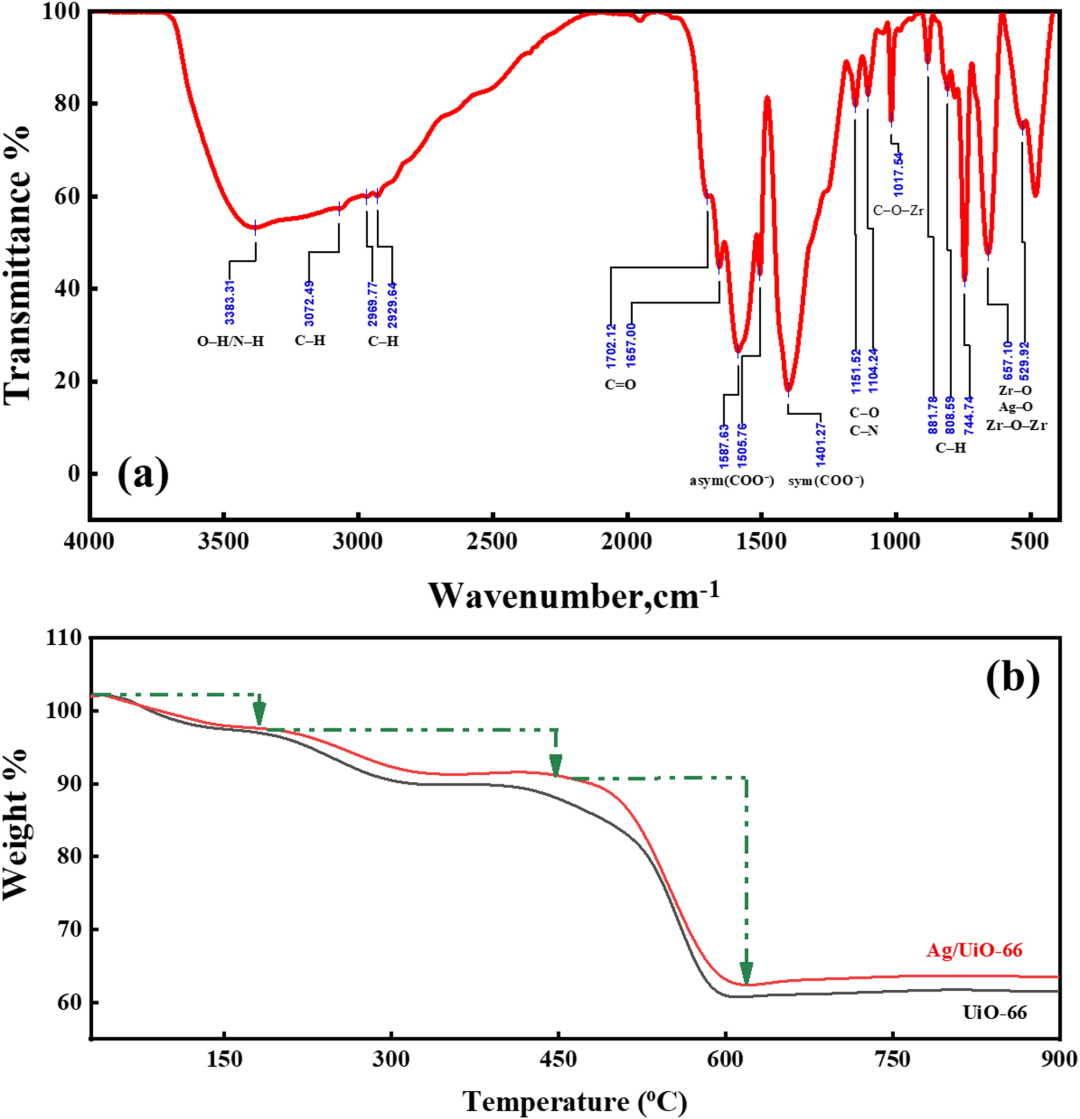
(**a**) FTIR spectrum of the Ag/UiO-66 composite and (**b**) TGA curves of UiO-66 and Ag/UiO-66.

The FTIR spectrum of Ag/UiO-66 (Fig. 2a) showed the characteristic vibration bands of the UiO-66 framework, which indicates that the framework was preserved after silver loading^60^. A broad band at about 3383 cm^−1^ is assigned to O–H stretching. This O–H signal comes from physisorbed water, terminal hydroxyl groups, and the µ-OH groups of the Zr_6_ clusters, and its broad shape reflects hydrogen bonding among these species. The bands at 3072, 2960, and 2929 cm^−1^ are assigned to C–H stretching of the aromatic ring of the terephthalate (BDC) linker. The band at 1702 cm^−1^, with a shoulder at 1657 cm^−1^, is assigned to C=O stretching and is linked to a small amount of protonated or free carboxyl groups at defect sites. The strong bands at 1587 and 1505 cm^−1^ correspond to the asymmetric stretching of the carboxylate groups (COO^−^), and the band at 1401 cm^−1^ corresponds to their symmetric stretching. The presence of both asymmetric and symmetric COO^−^ bands indicates that the carboxylate groups of the BDC linker are coordinated to the Zr nodes, consistent with the expected bonding in the UiO-66 framework. The bands at 1151 and 1104 cm^−1^ are assigned to C–O stretching, and the band at 1018 cm^−1^ to C–O–Zr vibrations, which further support the linker–node connection. The bands at 882, 808, and 745 cm^−1^ are assigned to aromatic C–H out-of-plane bending of the benzene ring of the linker. Finally, the bands at 657 and 630 cm^−1^ are assigned to Zr–O and Ag–O stretching, respectively, and the bands below 600 cm^−1^ to the Zr–O–Zr vibrations of the Zr_6_O_4_(OH)_4_ cluster^35,61^. The overall FTIR pattern of Ag/UiO-66 matched that of pristine UiO-66, with no new bands from impurities or by-products. This confirms that the structure of the linker and the Zr_6_ nodes was retained after silver loading and reduction.

The thermal stability of UiO-66 and Ag/UiO-66 was studied by TGA. The TGA curves (Fig. 2b) showed three main stages of weight loss. In the first stage, from room temperature to about 180 °C, the weight loss is due to the removal of physisorbed water and solvent molecules from the pores. In the second stage, from about 180 to 450 °C, the gradual weight loss is due to the loss of coordinated solvent (DMF) and the dehydroxylation of the Zr_6_ nodes. In the third stage, from about 450 to 620 °C, the sharp weight loss is due to the decomposition of the organic (BDC) linkers and the collapse of the framework, which leaves zirconium oxide (ZrO_2_) as the final residue. Both UiO-66 and Ag/UiO-66 exhibited similar thermal behavior, confirming that silver loading did not reduce the framework’s thermal stability. Ag/UiO-66 showed a higher residual mass than UiO-66, by about 3%. This higher residue is due to the silver species that remain after the framework decomposes, and it is consistent with the silver content measured by ICP-OES (3.11 wt%)^62^.

**Fig. 3:**
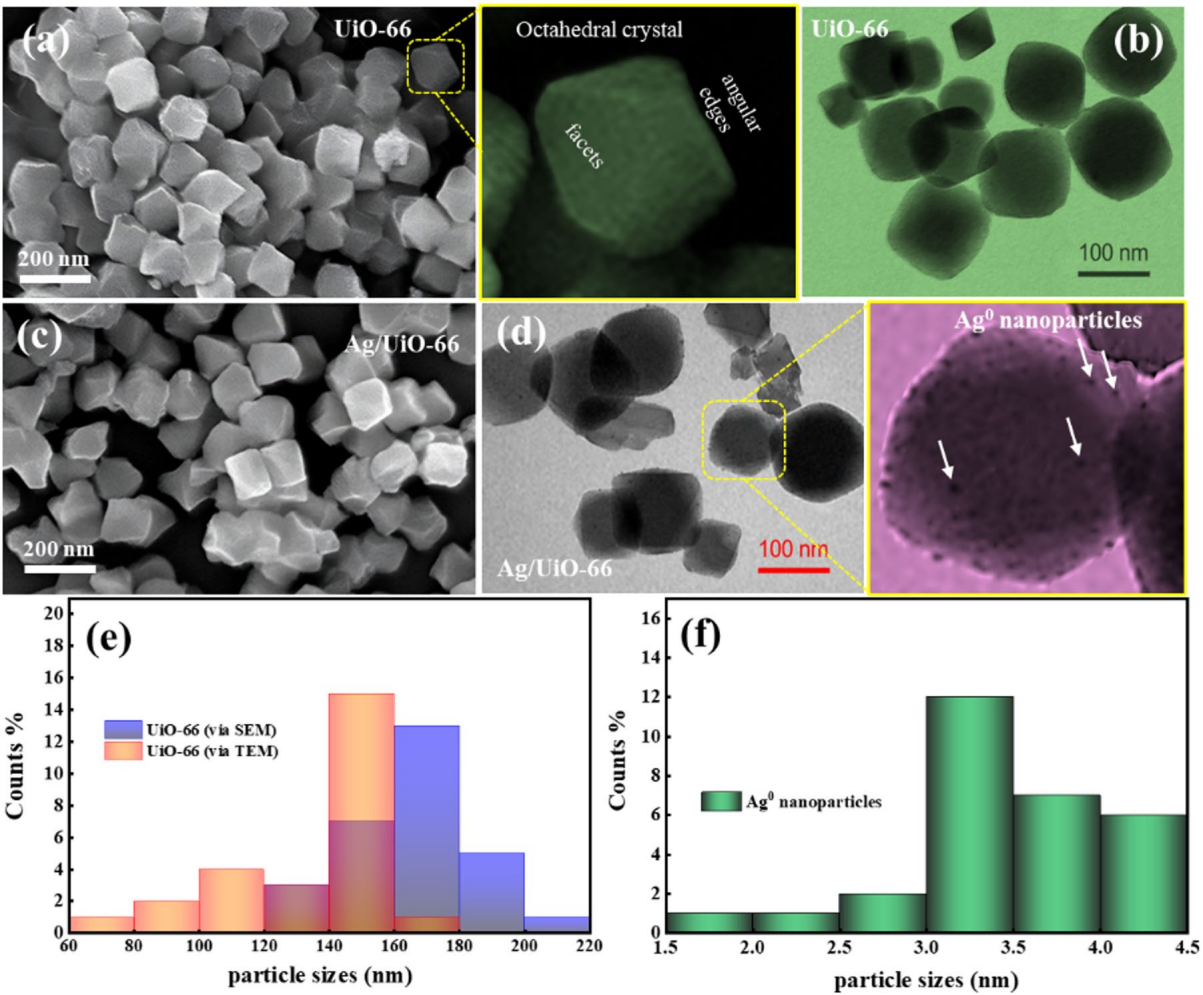
SEM and TEM characterization of the materials: (**a**) SEM image of UiO-66, with an inset showing an octahedral crystal; (**b**) TEM image of UiO-66; (**c**) SEM image of Ag/UiO-66; (**d**) TEM image of Ag/UiO-66, with a magnified inset showing the Ag⁰ nanoparticles (arrows); (**e**) particle-size distribution of UiO-66 from SEM and TEM images; and (**f**) particle-size distribution of the Ag⁰ nanoparticles from TEM images.

The morphology and particle size of UiO-66 and Ag/UiO-66 were studied by SEM and TEM^63^. The SEM image of UiO-66 (Fig. 3a) showed well-defined octahedral crystals with smooth facets and sharp edges, which is typical of UiO-66 prepared by a modulated synthesis. The magnified inset in Fig. 3a shows a single octahedral crystal with clear facets and angular edges. The TEM image of UiO-66 (Fig. 3b) confirmed the same octahedral shape and revealed crystals with uniform contrast, indicating a solid, well-formed framework. After silver loading, the SEM image of Ag/UiO-66 (Fig. 3c) showed that the crystals retained the same octahedral shape and size, indicating that the framework morphology was not damaged during the impregnation and reduction steps. The TEM image of Ag/UiO-66 (Fig. 3d) also retained the crystals’ octahedral shape. In addition, small dark spots were observed on and within the crystals, which were absent in pristine UiO-66. The magnified inset of Fig. 3d shows these dark spots more clearly (see arrows). The strong contrast between the dark spots and the lighter UiO-66 crystals is due to the higher electron density of silver compared with the Zr–organic framework. These dark spots are therefore assigned to metallic Ag⁰ nanoparticles formed after the NaBH₄ reduction step, and they appear well dispersed over the UiO-66 crystals.

The particle size of UiO-66 was measured from the SEM and TEM images using ImageJ software (Fig. 3e). The crystal size was in the range of about 140–165 nm. TEM gave a smaller average size of about 140 nm, while SEM gave a larger value of about 165 nm. This slight difference is typical because TEM measures well-defined, sharp-edged crystals, while SEM often captures surface features and small clusters, resulting in slightly higher values. The size distribution of the Ag⁰ nanoparticles was also measured from the TEM images (Fig. 3f). The nanoparticles ranged from about 1.5 to 4.5 nm, with most between 3 and 4 nm, and a mean size of about 3.3 nm. This narrow size distribution indicates that the silver was reduced to small, uniform nanoparticles that were well dispersed on the UiO-66 crystals. The small size and uniform dispersion of the Ag⁰ nanoparticles are useful for adsorption, because they provide more accessible active sites.

**Fig. 4:**
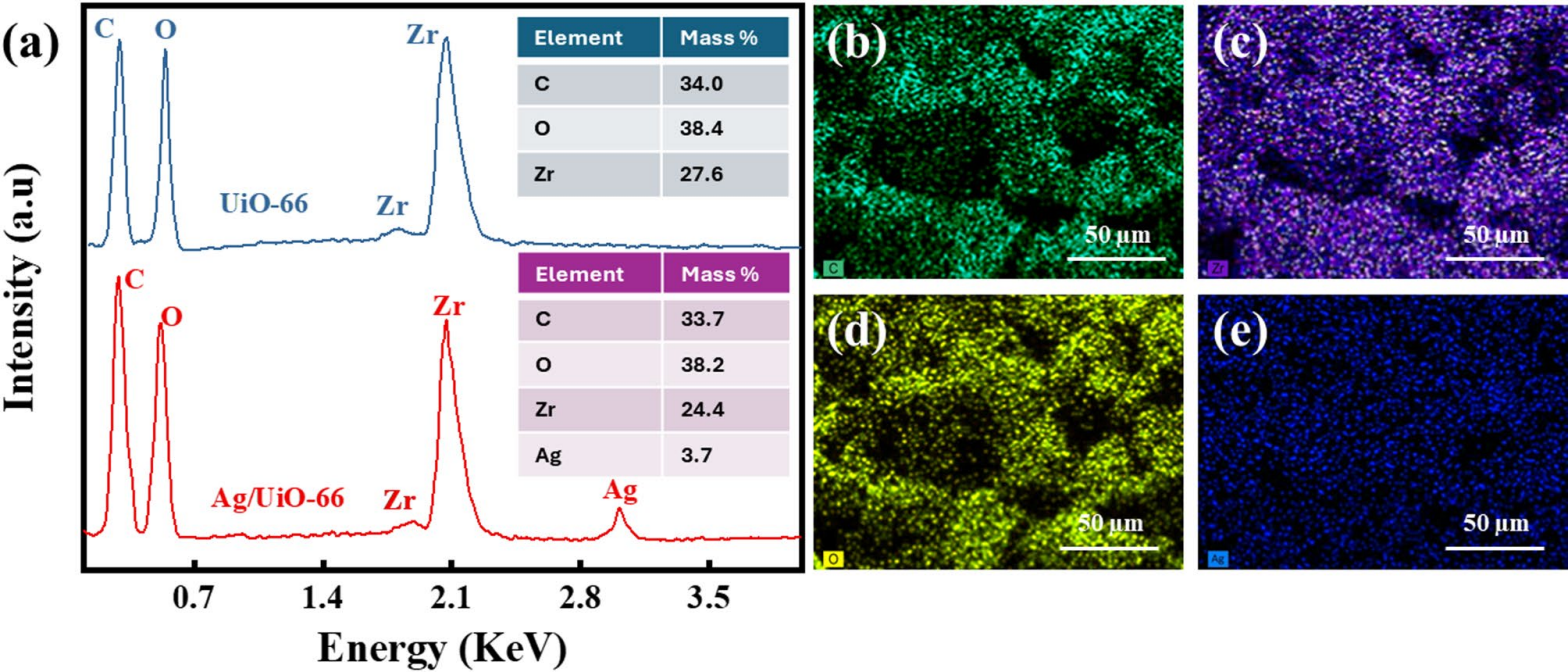
EDX analysis of the materials: (**a**) EDX spectra of UiO-66 and Ag/UiO-66, with the inset tables listing the elemental composition (mass %); and (**b**–**e**) EDX elemental maps of Ag/UiO-66 showing the distribution of (**b**) C, (**c**) Zr, (**d**) O, and (**e**) Ag.

The elemental composition and distribution of the samples were studied by EDX (Fig. 4a). The EDX spectrum of UiO-66 showed the emission lines of carbon (C Kα, ~ 0.277 keV), oxygen (O Kα, ~ 0.525 keV), and zirconium (Zr Lα, ~ 2.042 keV), which are the expected elements of the framework. The spectrum of Ag/UiO-66 showed the same lines, along with an additional silver line (Ag Lα, ~ 2.984 keV), confirming the presence of silver in the composite. The mass percentages from the inset table of Ag/UiO-66 were 33.7% for C, 38.2% for O, 24.4% for Zr, and 3.7% for Ag. The elemental maps of Ag/UiO-66 (Fig. 4b,e) showed that C, Zr, O, and Ag were evenly distributed over the crystals. In particular, the silver map (Fig. 4e) showed that the Ag was homogeneously dispersed, with no large clusters. This uniform distribution is consistent with the small, well-dispersed Ag⁰ nanoparticles observed by TEM. The silver content measured by ICP-OES was 3.11 wt%. This value is close to the EDX result but slightly lower because EDX is a semi-quantitative, surface-sensitive method, whereas ICP-OES measures the total silver content of the bulk sample. The ICP-OES value is therefore taken as the reliable measure of the composite’s silver content.

### MB and CV adsorption optimization

**Fig. 5:**
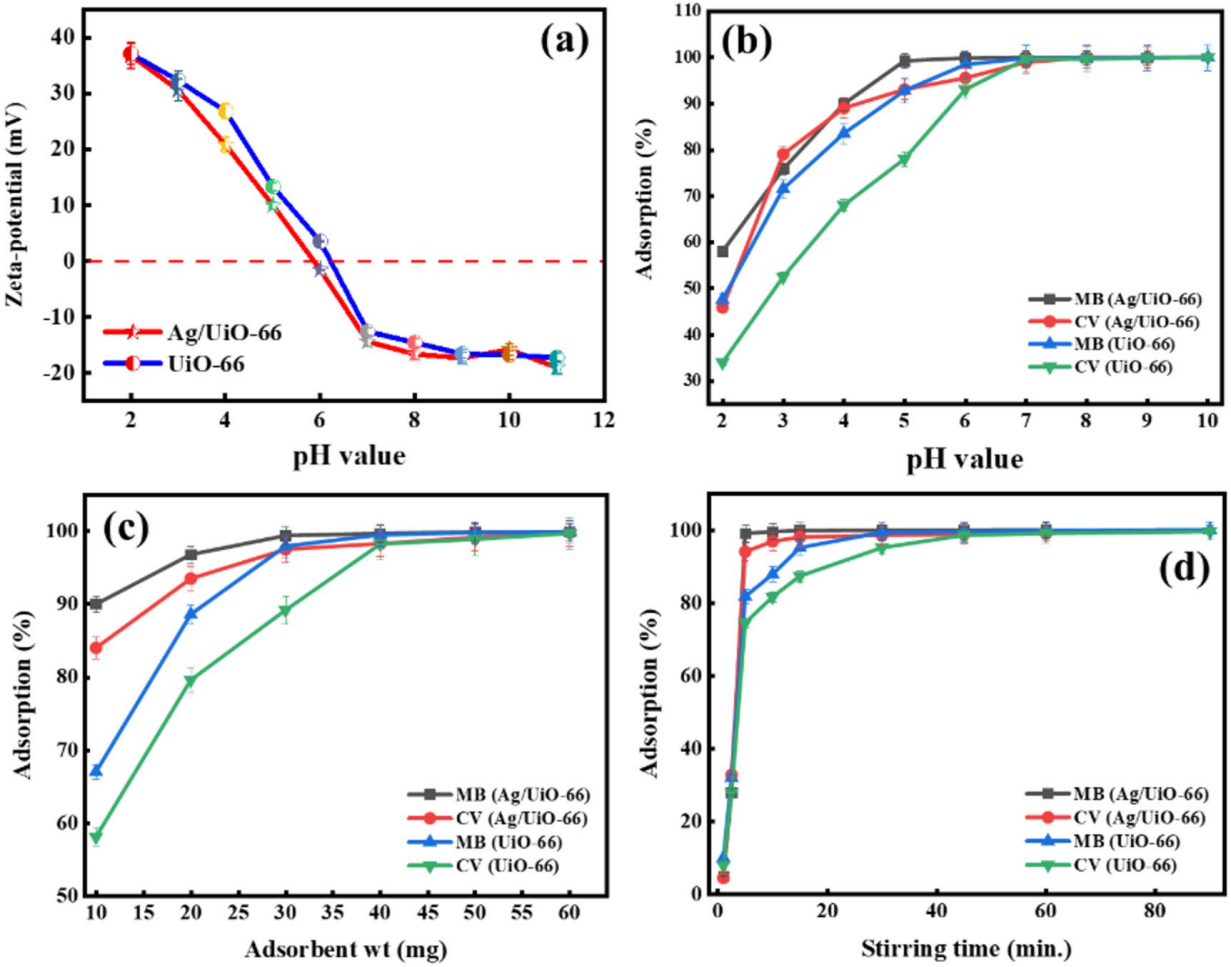
(**a**) Zeta potential of UiO-66 and Ag/UiO-66 as a function of pH; and adsorption performance of MB and CV on UiO-66 and Ag/UiO-66 under optimized conditions, showing the effect of (**b**) pH, (**c**) adsorbent dose, and (**d**) stirring time.

The surface charge of the materials was studied by zeta potential measurements in aqueous dispersions at 25 °C over a pH range of 2 to 11 (Fig. 5a). For both UiO-66 and Ag/UiO-66, the zeta potential was positive at low pH (about + 37 mV at pH 2), decreased as the pH increased, and became negative at high pH (about − 17 mV at pH 11). The Ag/UiO-66 curve was slightly more negative than that of UiO-66 over most of the pH range. The two curves crossed zero at different pH values, which correspond to their points of zero charge. The point of zero charge (pH_pzc_) was found to be 6.24 for UiO-66 and 5.88 for Ag/UiO-66, in good agreement with the zero-crossings of the zeta potential curves. The lower pH_pzc_ of Ag/UiO-66 shows that its surface becomes negative at a lower pH than that of UiO-66, which is consistent with its more negative zeta potential. For each material, the surface is positively charged below its pH_pzc_ and negatively charged above it. MB and CV are cationic dyes in water. Their adsorption is therefore expected to be favored above the pH_pzc_, because of the electrostatic attraction between the negatively charged surface and the positively charged dye molecules^64^.

The effect of pH on the removal of MB and CV by UiO-66 and Ag/UiO-66 was then studied over the pH range of 2 to 10 (Fig. 5b). For all four dye–adsorbent systems, the removal efficiency was lowest at pH 2 and increased quickly as the pH increased from 2 to 5. The removal then continued to rise more slowly up to pH 7, after which it stayed almost constant up to pH 10. This trend agrees with the surface charge behavior. At low pH, the surface is positively charged, and excess H^+^ ions compete with the cationic dye molecules for active sites, thereby reducing removal. As the pH rises above the pH_pzc_, the surface becomes negatively charged, and the electrostatic attraction between the surface and the cationic dyes increases, thereby increasing removal. At each pH, Ag/UiO-66 removed more dye than UiO-66, and MB was removed more than CV on both materials. The higher uptake of MB is attributed to its smaller molecular size, which facilitates diffusion into the pores and access to the active sites relative to the bulkier CV molecule. For all systems, the removal was highest and almost constant between pH 7 and pH 10. Neutral pH (pH 7) was therefore chosen as the optimum pH for the following experiments, because it gave near-maximum removal and is close to the pH of real wastewater, which is more practical for treatment.

The effect of adsorbent dose on the removal of MB and CV was studied using 10 to 60 mg of adsorbent at an initial dye concentration of 10 mg L^−1^, pH 7, and stirring at 300 rpm for 1 h in the dark (Fig. 5c). For all four dye–adsorbent systems, the removal efficiency increased quickly as the dose increased from 10 to 30 mg. This increase is due to the greater number of available active sites and the larger surface area provided by the additional adsorbent. Above 40 mg, the removal efficiency reached a plateau, indicating that most of the dye had already been removed and that adding more adsorbent provided little further benefit. Between 50 and 60 mg, the removal was nearly constant. At each dose, Ag/UiO-66 removed more dye than UiO-66, and MB was removed more than CV on both materials. This agrees with the trends observed in the pH study and with MB’s smaller molecular size, which facilitates access to the active sites relative to the larger CV molecule. Based on these results, 50 mg was chosen as the optimal dose for both dyes because it achieved near-maximum removal without excess adsorbent.

The effect of contact time on the removal of MB and CV by UiO-66 and Ag/UiO-66 was studied from 1 to 90 min under fixed conditions: C_o_ = 10 mg L^−1^, adsorbent dose = 50 mg, pH 7, 298 K, dark conditions, and stirring at 300 rpm (Fig. 5d). For all four systems, the adsorption was rapid, with most of the dye removed within the first 5–10 min, during which most of the dye was removed. The uptake then became slower and reached equilibrium within 45 min, after which the removal efficiency did not change. The fast initial step is due to the large number of free active sites on the fresh adsorbent. The slower step is due to the gradual filling of these sites, which lowers the driving force for further adsorption. At each contact time, Ag/UiO-66 reached equilibrium faster and removed more dye than UiO-66, and MB was removed faster than CV on both materials. This agrees with the trends observed in the pH and dose studies and with MB’s smaller molecular size. At equilibrium, both materials gave high removal efficiencies of more than 98% for both dyes. Therefore, 45 min was chosen as the optimum contact time for the following experiments.

### Adsorption kinetic investigations

The adsorption kinetics of MB and CV onto UiO-66 and Ag/UiO-66 were studied to elucidate the adsorption rate and potential rate-controlling steps. The experimental data were fitted with four common kinetic models: the pseudo-first-order (PFO), pseudo-second-order (PSO), Elovich, and intra-particle diffusion (IPD) models^65^. These models describe adsorption behavior but cannot directly prove the adsorption mechanism. The linear forms of the four models are given in Eqs. (3–6):

3 $$\:\begin{array}{c}Ln\left({\mathrm{q}}_{\mathrm{e}}-{\mathrm{q}}_{\mathrm{t}}\right)=Ln{\:\mathrm{q}}_{\mathrm{e}}-{\mathrm{k}}_{1}t \end{array}$$

4 $$\:\begin{array}{c}\frac{\mathrm{t}}{{\mathrm{q}}_{\mathrm{t}}}=\frac{\mathrm{t}}{{\mathrm{q}}_{\mathrm{e}}}+\frac{1}{{\mathrm{k}}_{2}{\mathrm{q}}_{\mathrm{e}}^{2}} \end{array}$$

5 $$\:\begin{array}{c}{\mathrm{q}}_{\mathrm{t}}=\frac{1}{{\upbeta\:}}\mathrm{ln}\left({\upalpha\:}{\upbeta\:}\right)+\frac{1}{{\upbeta\:}}\mathrm{ln}\left(\mathrm{t}\right) \end{array}\:\:\:\:\:\:$$

6 $$\:\begin{array}{c}{\mathrm{q}}_{\mathrm{t}}={\mathrm{k}}_{\mathrm{i}\mathrm{d}}{\mathrm{t}}^{0.5}+I \end{array}\:\:\:$$

where q_e_ and q_t_ (mg g^−1^) are the amounts of dye adsorbed at equilibrium and at time t. k_1_ (min^−1^) is the PFO rate constant, k_2_ (g mg^−1^ min^−1^) is the PSO rate constant, α (mg g^−1^ min^−1^) is the initial adsorption rate, and β (g mg^−1^) is the desorption constant of the Elovich model. k_id_ (mg g^−1^ min^−0.5^) is the IPD rate constant, and I (mg g^−1^) is a constant related to the thickness of the boundary layer.

**Fig. 6:**
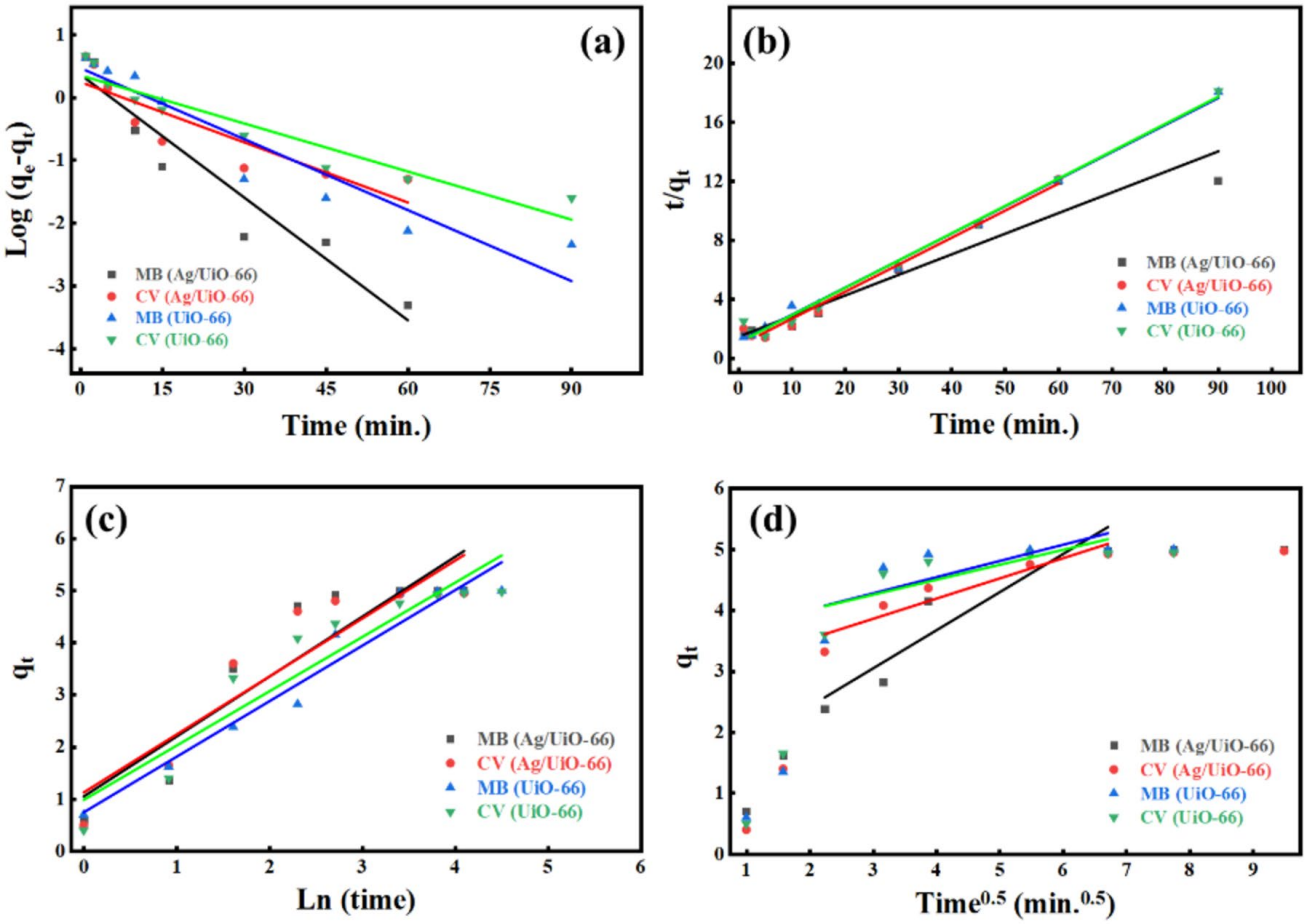
Linear fits of MB and CV adsorption on UiO-66 and Ag/UiO-66 using the (**a**) PFO, (**b**) PSO, (**c**) Elovich, and (**d**) IPD models.

The PFO model assumes that the adsorption rate is proportional to the number of free active sites. The PFO parameters were obtained from the linear plot of log(q_e_ − q_t_) versus t (Fig. 6a). As shown in Table 1, the PFO model gave moderate correlation coefficients (R^2^ = 0.781–0.929). More importantly, the q_e_ values calculated from the PFO model (1.28–1.60 mg g^−1^) were much lower than the experimental q_e_ values (about 4.95–4.99 mg g^−1^). This large discrepancy indicates that the PFO model does not adequately describe the adsorption of MB and CV.

The PSO model assumes that the adsorption rate depends on the square of the number of available active sites; although this is sometimes associated with chemical interactions, the model itself is a rate expression and does not confirm the adsorption mechanism. The PSO parameters were obtained from the linear plot of t/q_t_ versus t (Fig. 6b). The PSO model gave high correlation coefficients (R^2^ = 0.985–0.996) for all four systems. In addition, the q_e_ values calculated from the PSO model (5.44–5.52 mg g^−1^) were close to the experimental values. The PSO model therefore provided the best kinetic description of the data, indicating that the adsorption rate depends on the availability of active sites on the adsorbent. We note that a good PSO fit reflects the kinetic behavior of the system and does not, by itself, establish a chemisorption mechanism^66–69^.

The Elovich model is often used to describe adsorption on heterogeneous surfaces. The Elovich parameters were obtained from the linear plot of q_t_ versus ln t (Fig. 6c). The model yielded correlation coefficients of R^2^ = 0.847–0.950 (Table 1), indicating a reasonable but weaker fit than the PSO model^70^. The fit indicates that the adsorbent surface is energetically heterogeneous, which is consistent with the defect-rich structure of the modulated UiO-66. The IPD model was used to determine whether diffusion within the pores controls the adsorption rate. The IPD parameters were obtained from the plot of q_t_ versus t^0.5^ (Fig. 6d). The R^2^ values were low (0.51–0.85), which shows that IPD alone does not control the adsorption rate^71^. The plots did not pass through the origin, and the nonzero intercepts (I) indicate that boundary-layer (film-diffusion) effects also affect adsorption. These results indicate that adsorption occurs in more than one step: a rapid uptake on the outer surface in the first few minutes (about 2.5–5 min), followed by slower diffusion of the dye into the pores, and finally the approach to equilibrium^72^.

**Table 1:** Parameters of linear kinetic fitting of PFO, PSO, IPD, and Elovich models for MB and CV adsorption on UiO-66 and Ag/UiO-66 sorbents.

		UiO-66		Ag/UiO-66	
		MB	CV	MB	CV
PFO	q_e_ (mg g^− 1^)	1.597	1.422	1.440	1.278
	k_1_ (min^− 1^)	0.038	0.026	0.065	0.032
	R^2^	0.904	0.906	0.929	0.781
	RMSE	0.426	0.399	0.403	0.270
PSO	q_e_ (mg g^− 1^)	5.441	5.400	5.520	5.456
	k_2_ (g mg^− 1^ min^− 1^)	0.031	0.033	0.040	0.041
	R^2^	0.996	0.990	0.986	0.985
	RMSE	1.300	0.539	0.382	0.603
IPD	K_id (_mg g^−1^**min**^− 0.5^**)**	0.5	0.27	0.57	0.51
	I (mg g^− 1^)	1.63	3.08	1.89	2.11
	R^2^	0.8215	0.8483	0.5832	0.5136
	RMSE	0.7807	1.04117	1.1756	1.13669
Elovich	α (mg g^−1^**min**^− 1^)	0.9383	0.9596	0.8697	0.8991
	β (g mg^− 1^)	2.145	2.6769	2.8648	3.0623
	R^2^	0.9502	0.8902	0.847	0.8534
	RMSE	0.76039	0.71716	0.39801	0.59677

### Adsorption isotherm investigations

**Fig. 7:**
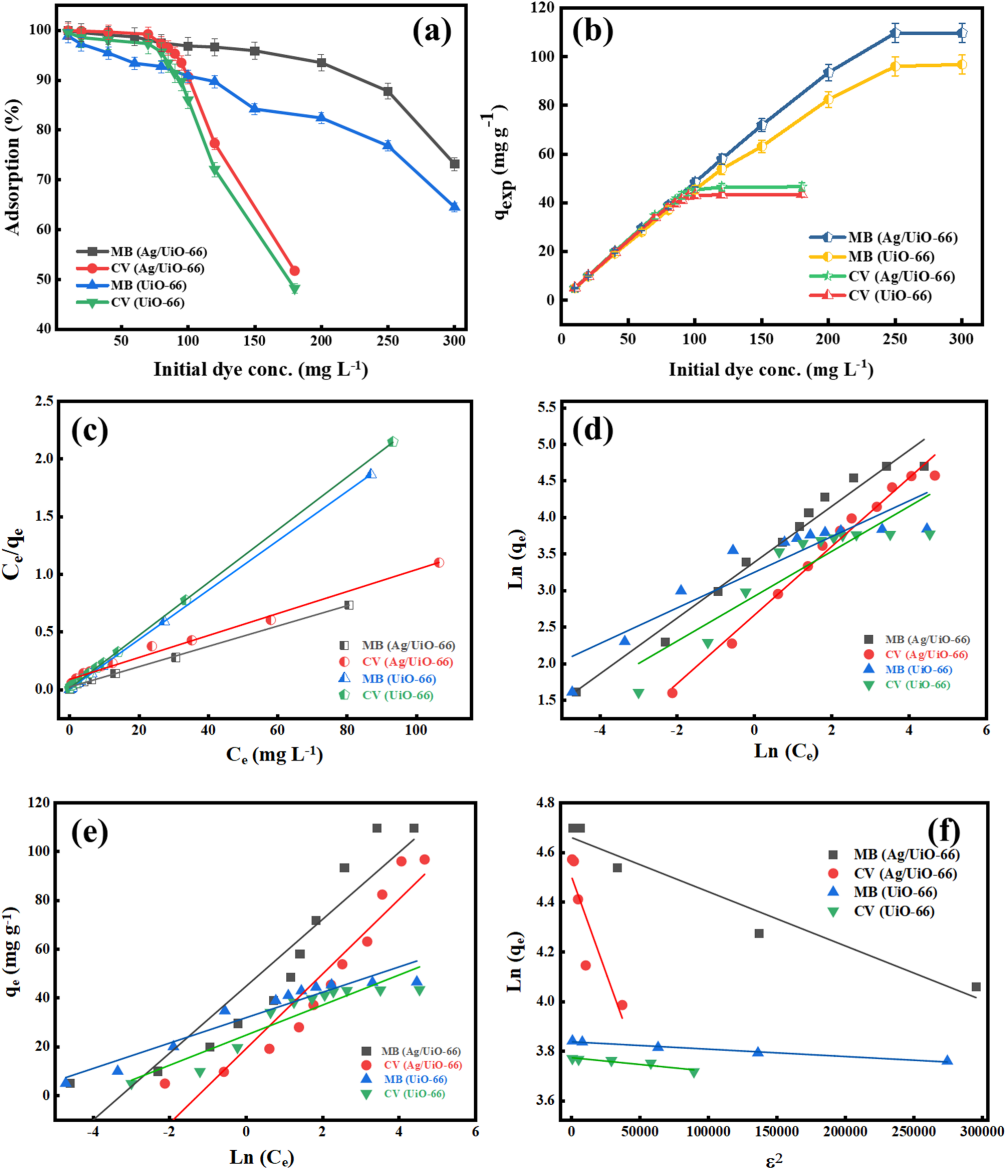
Effect of initial dye concentration on the (**a**) removal efficiency and (**b**) adsorption capacity of MB and CV on UiO-66 and Ag/UiO-66; and isotherm fits for MB and CV adsorption on UiO-66 and Ag/UiO-66 using the (**c**) Langmuir, (**d**) Freundlich, (**e**) Temkin, and (**f**) D–R models.

The effect of the initial dye concentration on the adsorption of MB and CV by UiO-66 and Ag/UiO-66 was studied over the range of 10 to 300 mg L^−1^ at pH 7 and 298 K, using a fixed adsorbent dose of 50 mg. Figure 7a shows the removal efficiency, and Fig. 7b shows the experimental adsorption capacity (q_exp_). As shown in Fig. 7a, both materials gave nearly complete removal at low concentrations (≤ 40 mg L^−1^), because the number of active sites was much larger than the number of dye molecules. As the concentration increased, the removal efficiency decreased, because the active sites became gradually filled. The decrease was clear at high concentrations (> 200 mg L^−1^), where the sites were almost saturated. At each concentration, MB was removed more than CV, and Ag/UiO-66 removed more than UiO-66. Figure 7b shows the opposite trend for the adsorption capacity. The q_exp_ increased with initial concentration because a higher concentration provided a stronger driving force, pushing more dye molecules onto the surface. At low concentrations, the q_exp_ curves for all four systems were nearly identical and rose in a straight line because almost all the dye had been removed. At higher concentrations, the curves separated and reached a plateau, indicating saturation of the active sites. The plateau values give the maximum experimental capacities. For MB, the q_exp_ reached about 109.7 mg g^−1^ on Ag/UiO-66 and 96.8 mg g^−1^ on UiO-66. For CV, the q_exp_ reached about 46.6 mg g^−1^ on Ag/UiO-66 and 43.4 mg g^−1^ on UiO-66. Two clear trends are evident in the capacity data. First, both dyes were adsorbed more on Ag/UiO-66 than on UiO-66, suggesting that Ag incorporation may introduce additional interfacial adsorption regions that enhance uptake. Second, MB was adsorbed much more than CV on both materials. As noted above, this trend reflects the smaller molecular size of MB, which favours its diffusion into the framework pores compared with CV.

Adsorption isotherms describe the relationship between the amount of dye adsorbed and the equilibrium concentration in solution at a fixed temperature. Fitting the equilibrium data to isotherm models gives information about the maximum capacity and the type of adsorption^73,74^. These models are simplified descriptions and cannot directly prove the mechanism of adsorption. The equilibrium data for MB and CV on UiO-66 and Ag/UiO-66 were fitted with four common isotherm models: the Langmuir, Freundlich, Temkin, and Dubinin–Radushkevich (D–R) models. The linear forms of these models, and the related equations, are given in Eqs. (7–14):

7 $$\:\begin{array}{c}\frac{{\mathrm{C}}_{\mathrm{e}}}{{\mathrm{q}}_{\mathrm{e}}}=\frac{1}{{\mathrm{K}}_{\mathrm{L}}{\mathrm{q}}_{\mathrm{m}}}+\left(\frac{1}{{\mathrm{q}}_{\mathrm{m}}}\right){\mathrm{c}}_{\mathrm{e}} \end{array}$$

8 $$\:\begin{array}{c}{\mathrm{R}}_{\mathrm{L}}=\frac{1}{1+{\mathrm{C}}_{0}{\mathrm{K}}_{\mathrm{L}}} \end{array}$$

9 $$\:\begin{array}{c}\:\mathrm{ln}{\mathrm{q}}_{\mathrm{e}}=\mathrm{ln}{\mathrm{K}}_{\mathrm{f}}\:+\:\frac{1}{\mathrm{n}}\:\mathrm{ln}{\mathrm{c}}_{\mathrm{e}\:} \end{array}$$

10 $$\:\begin{array}{c}{\mathrm{q}}_{\mathrm{e}}=Bln{\mathrm{K}}_{\mathrm{T}}+Bln{\mathrm{c}}_{\mathrm{e}} \end{array}$$

11 $$\:\begin{array}{c}B=\frac{\mathrm{R}\mathrm{T}}{\mathrm{b}} \end{array}$$

12 $$\:\begin{array}{c}\:ln{\mathrm{q}}_{\mathrm{e}}=ln{\mathrm{q}}_{\mathrm{o}}-\delta\:{\varepsilon}^{2} \end{array}$$

13 $$\:\begin{array}{c}\varepsilon=RTln\left(1+\frac{1}{{\mathrm{C}}_{\mathrm{e}}}\right) \end{array}$$

14 $$\:\begin{array}{c}E=\frac{1}{\sqrt{2{\updelta\:}}} \end{array}$$

For the Langmuir model, q_m_ (mg g^−1^) is the maximum adsorption capacity, K_L_ (L mg^−1^) is the Langmuir constant, and R_L_ is the dimensionless separation factor. For the Freundlich model, K_f_ (mg g^−1^) is related to the adsorption capacity, and n is related to the adsorption intensity. For the Temkin model, B (J mol^−1^) is related to the heat of adsorption, b is the Temkin constant, and K_T_ (L g^−1^) is the Temkin isotherm constant. For the D–R model, q_o_ (mg g^−1^) is the maximum capacity, δ (mol^2^ J^−2^) is a constant related to the adsorption energy, ε is the Polanyi potential, and E (kJ mol^−1^) is the mean free energy of adsorption. T is the absolute temperature (K), and R is the gas constant (8.314 J mol^−1^ K^−1^).

The Langmuir model assumes monolayer adsorption on a uniform surface with identical and independent active sites^75,76^. The values of q_m_ and K_L_ were obtained from the linear plot of C_e_/q_e_ versus C_e_ (Fig. 7c). All four systems gave excellent Langmuir fits, with R^2^ > 0.98 (Table 2). This shows that the Langmuir model describes the equilibrium data well and that the adsorption is mainly monolayer on a uniform surface. After silver loading, the q_m_ increased from 104.98 to 113.49 mg g^−1^ for MB and from 43.72 to 46.72 mg g^−1^ for CV. These q_m_ values are close to the experimental capacities, which confirms that the Langmuir fit is reliable. The favorability of the adsorption was also checked using the separation factor R_L_. The value of R_L_ shows the type of adsorption: R_L_ > 1 is unfavorable, R_L_ = 1 is linear, 0 < R_L_ < 1 is favorable, and R_L_ = 0 is irreversible. All systems yielded R_L_ values between 0 and 1, confirming that the adsorption of MB and CV was favorable.

The Freundlich model assumes multilayer adsorption on a heterogeneous surface with sites of different energies. The values of K_f_ and n were obtained from the linear plot of ln q_e_ versus ln C_e_ (Fig. 7d). All systems showed n values greater than 1, indicating favorable adsorption. The Freundlich model fitted the MB data well (R^2^ = 0.973–0.984) but fitted the CV data less well (R^2^ = 0.818–0.838). Overall, the Freundlich fits were weaker than the Langmuir fits, which supports monolayer rather than multilayer adsorption.

The Temkin model assumes that the heat of adsorption of all molecules decreases linearly with surface coverage, due to adsorbate–adsorbent interactions. The parameters were obtained from the linear plot of q_e_ versus ln C_e_ (Fig. 7e). As shown in Table 2, the Temkin model gave lower R^2^ values than the Langmuir model for all systems, so it gave a less satisfactory description of the equilibrium data. The D–R model was used to estimate the mean free energy of adsorption (E) and to find out whether the adsorption is physical or chemical. As a guide, E below 8 kJ mol^−1^ indicates physical adsorption, E between 8 and 16 kJ mol^−1^ indicates ion exchange, and E above 16 kJ mol^−1^ indicates chemisorption. The parameters were obtained from the plot of ln q_e_ versus ε^2^ (Fig. 7f). As shown in Table 2, all systems gave E values below 2 kJ mol^−1^. These low E values indicate that physical interactions contribute to the adsorption. However, because the D–R fits were weaker than the Langmuir fits for some systems, the E values are taken as indicative rather than exact. This physical contribution, together with the good fit of the Langmuir isotherm and the pseudo-second-order kinetic model, shows that the adsorption of MB and CV involves both physical and chemical interactions^77^. The presence of a physical contribution is also consistent with the low heat of adsorption obtained from the thermodynamic study.

**Table 2:** Parameters of Langmuir, Freundlich, Temkin, and D-R isotherm models for MB and CV adsorption on UiO-66 and Ag/UiO-66 sorbents.

		UIO-66		Ag/UIO-66	
		MB	CV	MB	CV
Exp. capacity	q_exp_ (mg g^− 1^)	96.8	43.4	109.7	46.6
Langmuir	q_max_ (mg g^− 1^)	104.98	43.72	113.49	46.72
	K_L_ (L mg^− 1^)	0.11	1.82	0.4	3.79
	R_L_	< 1	< 1	< 1	< 1
	R^2^	0.9873	0.9999	0.9968	0.9999
Freundlich	1/n	0.47	0.31	0.38	0.24
	n	2.13	3.22	2.63	4.16
	K_f_ (mg g^− 1^)	14.35	18.56	29.57	25.76
	R^2^	0.9836	0.818	0.973	0.8377
Temkin	B_t_	15.315	6.168	13.717	5.207
	b	161.851	401.86	180.714	476.04
	K_t_ (L mg^− 1^)	3.499	55.298	26.165	458.631
	R^2^	0.9001	0.8626	0.8741	0.9192
D-R	δ (mol^2^ J^− 2^)	1.54E-05	5.47E-07	2.18E-06	2.96E-07
	q_o_ (mg g^− 1^)	90.402	43.545	105.65	46.414
	E (kJ mol^− 1^)	0.18	0.955	0.478	1.299
	R^2^	0.7952	0.8929	0.9489	0.9866

### Thermodynamic studies

**Fig. 8:**
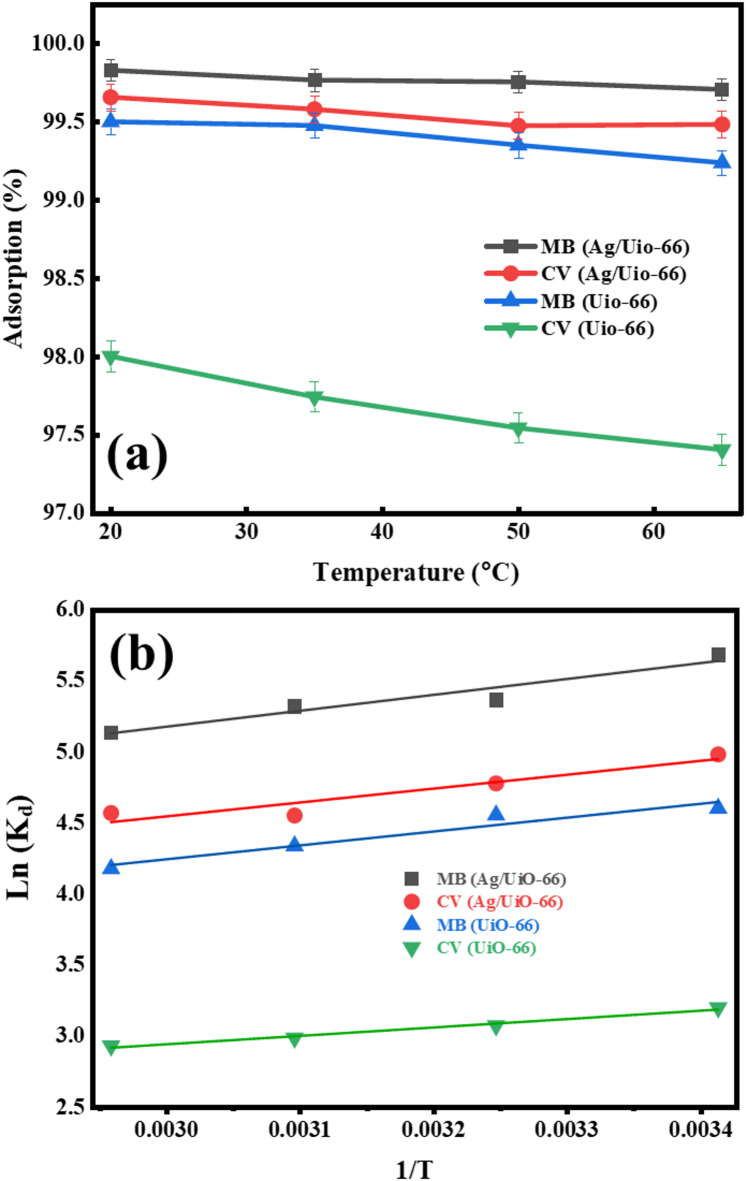
(**a**) Effect of temperature on the adsorption of MB and CV on UiO-66 and Ag/UiO-66; and (**b**) Van’t Hoff plots (ln K_d_ vs. 1/T) for the same systems.

Temperature affects the adsorption process by altering the interactions between the dye and the adsorbent and the movement of the dye molecules. The effect of temperature on the removal of MB and CV by UiO-66 and Ag/UiO-66 was studied over the range of 20 to 65 °C (298 to 338 K). As shown in Fig. 8a, the removal efficiency of both dyes decreased slightly as the temperature increased. This decrease shows that the adsorption is exothermic. To understand the thermodynamic nature of the process, the equilibrium data were analyzed using the Van’t Hoff method. The distribution coefficient (K_d_) was calculated at each temperature, and the standard thermodynamic parameters were obtained from the plot of ln K_d_ versus 1/T (Fig. 8b) using Eqs. (15–18):

15 $$\:\begin{array}{c}\varDelta\:G^\circ\:=-RTLn{\mathrm{K}}_{\mathrm{d}} \end{array}$$

16 $$\:\begin{array}{c}\varDelta\:G^\circ\:=\varDelta\:H^\circ\:-T\varDelta\:S^\circ \end{array}$$

17 $$\:\begin{array}{c}Ln{\mathrm{K}}_{\mathrm{d}}=\frac{\varDelta\:\mathrm{S}^\circ\:}{\mathrm{R}}-\frac{\varDelta\:\mathrm{H}^\circ\:}{\mathrm{R}\mathrm{T}} \end{array}$$

18 $$\:\begin{array}{c}{\mathrm{K}}_{\mathrm{d}}=\frac{{\mathrm{q}}_{\mathrm{e}}}{{\mathrm{c}}_{\mathrm{e}}} \end{array}$$

where ΔG° (kJ mol^−1^), ΔH° (kJ mol^−1^), and ΔS° (J mol^−1^ K^−1^) are the changes in Gibbs free energy, enthalpy, and entropy, respectively. The values of ΔH° and ΔS° were obtained from the slope and intercept of the ln K_d_ versus 1/T plot, and ΔG° was calculated at each temperature. The calculated parameters are summarized in Table 3. The ΔH° values were negative for all systems (− 4.91 to − 9.31 kJ mol^−1^), which confirms that the adsorption is exothermic. This agrees with the decrease in removal efficiency at higher temperatures (Fig. 8a) and with the positive slope of the Van’t Hoff plots (Fig. 8b). The small magnitude of ΔH° indicates that the adsorption is physical, which is consistent with the low adsorption energy obtained from the D–R model. The ΔS° values were positive for all systems (9.74 to 15.12 J mol^−1^ K^−1^), which indicates an increase in randomness at the solid–solution interface during adsorption. This increase is likely due to the release of water molecules from the hydration shells of the dye molecules and from the adsorbent surface into the solution. The ΔG° values were negative across all temperatures, indicating that the adsorption of MB and CV is spontaneous and thermodynamically favorable.

**Table 3:** Thermodynamic parameters (ΔG°, ΔH°, ΔS°) for MB and CV adsorption on UiO-66 and Ag/UiO-66 over 298–338 K.

		Temp. (K)	∆G° (KJ mol^− 1^)	∆H° (KJ mol^− 1^)	∆S° (J mol^−1^K^− 1^)	R^2^
UiO-66	MB	298	− 11.212	− 8.114	10.947	0.9351
		308	− 11.665			
		323	− 11.651			
		338	− 11.740			
	CV	298	− 7.796	− 4.912	9.739	0.9837
		308	− 7.876			
		323	− 8.0272			
		338	− 8.241			
Ag/UiO-66	MB	298	− 13.842	− 9.305	15.12	0.9309
		308	− 13.742			
		323	− 14.285			
		338	− 14.434			
	CV	298	− 12.136	− 8.125	13.42	0.8949
		308	− 12.234			
		323	− 12.225			
		338	− 12.841			

### Adsorption mechanism

The adsorption mechanism of MB and CV on Ag/UiO-66 was studied by comparing the material before and after adsorption using FTIR and XPS, and by considering the surface charge from the zeta potential measurements. The FTIR spectra of Ag/UiO-66 after MB and CV adsorption (Fig. S4) showed changes in several bands compared with the fresh material. Changes were seen in the broad O–H band at 3300–3500 cm^−1^, the aromatic C = C region near 1580–1600 cm^−1^, the carboxylate and C=O region at 1390–1700 cm^−1^, and the framework bands at 500–800 cm^−1^. These changes indicate that the hydroxyl groups, aromatic linkers, carboxylate groups, and metal–oxygen sites of the framework all participate in dye adsorption^54^. The XPS survey spectra gave further evidence for dye adsorption (Fig. S5). After MB adsorption, new N 1s and S 2p peaks appeared at about 400.1 and 165.2 eV. After CV adsorption, a new N 1s peak appeared at about 400.2 eV. The S 2p peak was observed only after MB adsorption because MB contains sulfur, whereas CV does not. These new peaks confirm that the dye molecules were adsorbed on the surface of Ag/UiO-66. The zeta-potential results account for the pH-dependent adsorption behaviour. At pH 7, which is above the point of zero charge of both UiO-66 (pH_pzc_ = 6.24) and Ag/UiO-66 (pH_pzc_ = 5.88), the surface of the adsorbent is negatively charged. MB and CV are cationic dyes. Therefore, there is a strong electrostatic attraction between the negatively charged surface and the positively charged dye molecules, which is one of the main driving forces of adsorption.

**Fig. 9:**
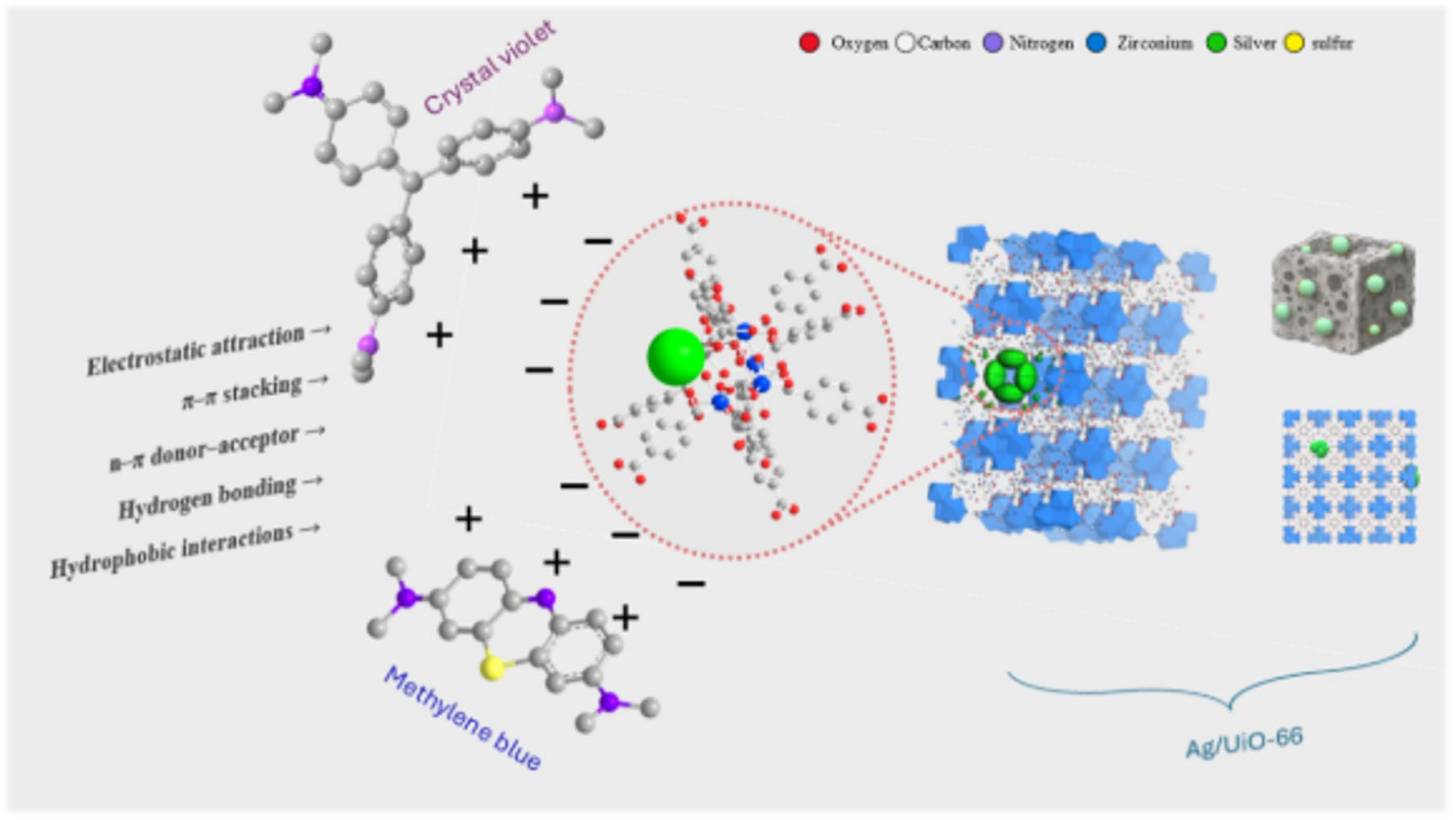
Schematic illustration of the proposed mechanism for MB and CV adsorption on Ag/UiO-66, showing electrostatic attraction, π–π stacking, n–π donor–acceptor interactions, hydrogen bonding, and hydrophobic interactions.

Several other interactions also contribute to the adsorption, as shown in Fig. 9. π–π stacking can occur between the aromatic rings of the dyes and the benzene rings of the terephthalate linkers. Hydrogen bonding can form between the dye molecules and the hydroxyl and other oxygen-containing groups of the framework, consistent with the FTIR changes in the O–H and carboxylate regions. n–π and hydrophobic interactions between the aromatic dye molecules and the framework can also take place. The low adsorption energy from the D–R model (below 2 kJ mol^−1^) and the low, negative ΔH° from the thermodynamic study show that physical interactions are present. The good fits of the PSO kinetic model and the Langmuir isotherm are consistent with adsorption occurring at a finite number of specific active sites. This shows that the adsorption of MB and CV on Ag/UiO-66 is not controlled by a single mechanism, but by both physical and chemical interactions acting together.

Although Ag/UiO-66 had a lower BET surface area than UiO-66, it showed higher adsorption capacities for both MB and CV. This shows that adsorption is controlled mainly by the number and strength of active sites, not by surface area alone. This is seen more clearly when the capacity is normalized to the BET surface area. For MB, the area-normalized capacity increases from 0.121 mg m^−2^ (104.98/870) for UiO-66 to 0.177 mg m^−2^ (113.49/642) for Ag/UiO-66, an increase of about 46%; for CV, it increases from 0.050 to 0.073 mg m^−2^, an increase of about 45%. Thus, per unit of accessible surface, the Ag-loaded material adsorbs substantially more dye, confirming that the enhancement is not a surface-area effect. The metallic Ag⁰ nanoparticles (about 3.11 wt%) in the defect-rich UiO-66 framework introduce additional active sites and alter the material’s surface chemistry. Because metallic Ag⁰ is not itself a high-capacity adsorbent, its role is best understood as providing localized high-affinity sites rather than additional surface area. The defect sites formed during HCl modulation may further increase the affinity for the dye molecules, although their contribution could not be separated from that of the silver in the present study. Both MB and CV are planar, aromatic, π-conjugated cations, and the appearance of new N 1s (both dyes) and S 2p (MB only) signals in the post-adsorption XPS confirms that the dyes are adsorbed onto the composite; these framework- and Ag-associated sites may participate in dye binding, although a silver-specific contribution is not directly demonstrated by these measurements. This explains why Ag/UiO-66 adsorbs more dye than UiO-66, despite its lower surface area. A similar behavior has been reported for other metal-loaded MOFs, in which the added guest–host interactions offset the small loss in surface area. It should be noted that the present study compared HCl-modulated UiO-66 with its Ag-loaded counterpart but did not include a non-modulated, defect-free reference. Consequently, the individual contributions of the HCl-generated defects and the silver nanoparticles to the enhanced uptake could not be quantitatively separated, and the proposed Ag-specific interactions, while consistent with the spectroscopic evidence, are not directly demonstrated. Distinguishing these effects would require dedicated experiments, including adsorption on pristine, defect-modulated, and Ag-free frameworks, adsorption on the silver nanoparticles alone, and analysis of Ag 3d peak shifts before and after dye uptake.

### Reusability of Ag/UiO-66 adsorbent

**Fig. 10:**
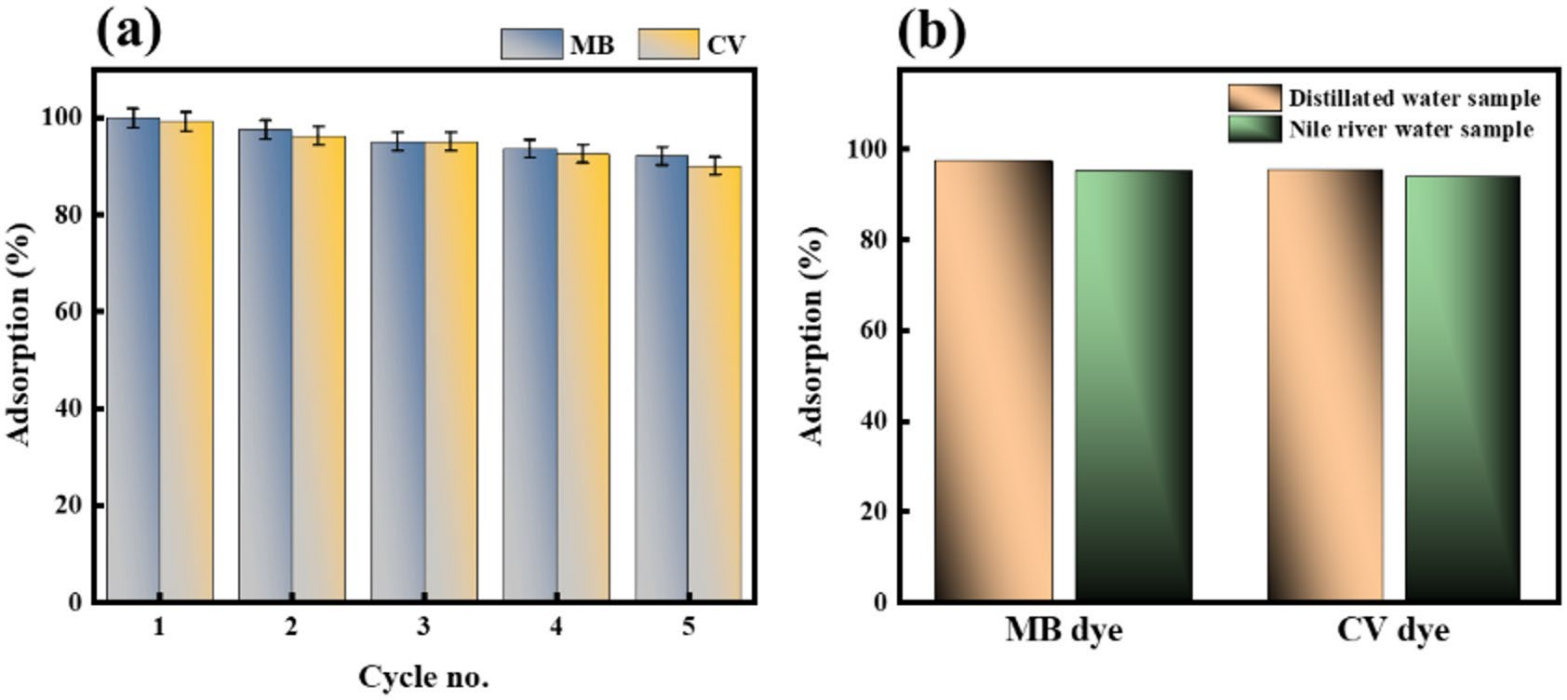
(**a**) Reusability of Ag/UiO-66 for MB and CV adsorption over five adsorption–desorption cycles; and (**b**) removal of MB and CV by Ag/UiO-66 from solutions prepared in distilled water and Nile River water.

The reusability of an adsorbent is an important factor for its use in real wastewater treatment. The regeneration of Ag/UiO-66 was tested for the removal of MB and CV over five adsorption–desorption cycles. In each cycle, 50 mg of adsorbent was mixed with 25 mL of a 10 mg L^− 1^ dye solution and stirred for 45 min at room temperature. After adsorption, the used adsorbent was regenerated by sonication in ethanol for 30 min (repeated twice), then washed with distilled water and dried at 80 °C. As shown in Fig. 10a, Ag/UiO-66 retained high reusability. The removal efficiency remained above 90% for both MB and CV over the five cycles. The efficiency decreased only slightly, from about 99% in the first cycle to about 90% in the fifth cycle. This small decrease shows that the material can be reused several times. The regeneration used only common solvents and mild conditions, which makes the process simple and lowers the need to replace the adsorbent often.

The stability of the material was also checked by PXRD after the fifth cycle (Fig. S6). The main diffraction peaks of UiO-66 were still present, indicating that the framework remained stable during the adsorption–desorption cycles. However, ICP-OES analysis showed that the silver content decreased from 3.11 wt% in the fresh composite to 2.64 wt% after one cycle. In agreement with this, dissolved silver was detected in the supernatant at a concentration of 0.41 mg L^− 1^. These results show that a small amount of silver leached from the composite during use. The loss of silver is one reason for the gradual decrease in adsorption efficiency over the cycles. Stronger stabilization of the Ag nanoparticles on the framework would reduce this leaching and improve the composite’s long-term stability.

### Application to a real water matrix

The practical applicability of Ag/UiO-66 was evaluated using surface water collected from the Nile River as a representative real water matrix. The physicochemical characteristics of the Nile water sample are summarized in Table 4. Dye solutions of MB and CV were prepared in the Nile water sample, and equivalent solutions were prepared in distilled water as a control under identical conditions. In each experiment, 50 mg of Ag/UiO-66 was added to 25 mL of dye solution (10 mg L^−1^) and stirred for 45 min at room temperature, after which the removal efficiency was determined. As shown in Fig. 10b, Ag/UiO-66 achieved removal efficiencies of 95.26% for MB and 93.58% for CV in the Nile water matrix, only marginally lower than the values obtained in distilled water. This limited reduction is notable given the presence of several co-existing species in the Nile water that could compete with the cationic dyes for the available adsorption sites, including a total hardness of 107 mg L^−1^, total dissolved solids of 157 mg L^−1^, and chlorides of 21 mg L^−1^ (Table 4). In particular, the divalent Ca^2+^ and Mg^2+^ cations associated with the water hardness represent potential competitors for the negatively charged surface sites of the adsorbent. The retention of high removal efficiencies in this competitive environment indicates that Ag/UiO-66 exhibits good selectivity toward the cationic dyes and maintains its adsorption performance under realistic conditions. These findings support the potential of Ag/UiO-66 as an effective adsorbent for treating dye-contaminated wastewater^33^.

**Table 4:** Physicochemical characteristics of the Nile water matrix used in the adsorption experiments.

Parameter	Concentration
Turbidity (NTU)	5.8
Total alkalinity (mg L^− 1^)	160
Total hardness (mg L^− 1^)	107
Chlorides (mg L^− 1^)	21
Calcium hardness (mg L^− 1^)	65
Magnesium hardness (mg L^− 1^)	42
Total dissolved solids (TDS) (mg L^− 1^)	157
Ammonia (mg L^− 1^)	0.13
Iron (mg L^− 1^)	0.11
Manganese (mg L^− 1^)	0.01

### Comparison with reported adsorbents

The adsorption performance of Ag/UiO-66 was compared with that of other adsorbents reported for the removal of MB and CV, as summarized in Table 5. The maximum adsorption capacity of Ag/UiO-66 was 113.49 mg g^−1^ for MB and 46.72 mg g^−1^ for CV. For MB, this capacity was higher than that of the other UiO-66- and MOF-based adsorbents in the comparison, including UiO-66/GO-COOH (54.96 mg g^−1^), NH_2_-UiO-66 (90 mg g^−1^), and MoS_2_/ZIF-8 (85.52 mg g^−1^). For CV, the capacity of Ag/UiO-66 was also higher than that of the reported adsorbents, such as potato-peel-derived carbon (29.5 mg g^−1^) and the volcanic ash/rice husk geopolymer (14.6 mg g^−1^). In addition to the higher capacity, Ag/UiO-66 removed both dyes efficiently at neutral pH (pH 7). Many of the reported adsorbents required alkaline conditions to achieve optimal performance, for example, pH 9 for NH_2_-UiO-66, pH 10 for the duckweed-derived adsorbent, and pH 11 for the treated kaolin. The ability of Ag/UiO-66 to operate at neutral pH is an advantage for real applications, as it avoids the extra cost and chemicals required to adjust wastewater pH. Ag/UiO-66 was also able to remove both a small-molecule cationic dye (MB) and a larger-molecule cationic dye (CV) using the same material. These results show that Ag/UiO-66 is an effective and practical adsorbent for removing cationic dyes from water.

**Table 5:** Comparison of removal of dyes by our adsorbent and reported adsorbents.

Adsorbent	Adsorbate	pH condition	q_max_ (mg g^−1^)	Ref.
UiO-66/GOCOOH	MB	7	54.96	^78^
NH_2_-UiO-66	MB	9	90	^79^
Starch-functionalized graphene oxide-polyethyleneimine composite	MB	9	6.85	^80^
MoS_2_/ZIF-8 composite	MB	7	85.52	^81^
Treated Kaolin	MB	11	64.58	^82^
Green magnetic duckweed-derived adsorbent	MB	10	78.59	^83^
Eggshell-derived CaO	CV	3	6.76	^84^
Volcanic ash/rice husk ash based phosphate geopolymer	CV	6	14.6	^85^
Kaolin-based geopolymer	CV	10.2	7.27	^86^
Potato peels-derived carbon	CV	7	29.5	^87^
Ag/UiO-66	MB	7 (neutral)	113.49	Here
Ag/UiO-66	CV	7 (neutral)	46.7	Here

## Conclusion

In this study, an Ag/UiO-66 composite was successfully prepared using silver recovered from electrical contact waste as the source, offering a circular-economy route to a functional adsorbent. Comprehensive characterization demonstrated the incorporation of well-dispersed metallic Ag⁰ nanoparticles (about 3.3 nm; 3.11 wt% by ICP-OES) while the crystalline UiO-66 framework was preserved. The composite showed enhanced adsorption of the cationic dyes (MB and CV), with Langmuir maximum capacities of 113.49 and 46.72 mg g^−1^, respectively, both higher than those of pristine UiO-66 (104.98 and 43.72 mg g^−1^). Under the optimized conditions, removal efficiencies above 98% were achieved within 45 min at neutral pH. The equilibrium data were best described by the Langmuir isotherm, and the kinetics were best described by the PSO model. The low adsorption energies in the D–R model and the negative enthalpy values from the thermodynamic study indicated that the uptake is spontaneous, exothermic, and proceeds via combined physical and chemical interactions. The zeta-potential and spectroscopic analyses further showed that electrostatic attraction, π–π stacking, hydrogen bonding, and interactions with the silver and defect sites all contribute to the adsorption. The composite also exhibited high reusability, retaining more than 90% removal over five cycles, and maintained high efficiency in a real water matrix, achieving 95.3% removal of MB and 93.6% of CV in Nile River water. These results demonstrate that Ag/UiO-66 prepared from recycled silver is an effective and practical adsorbent for removing cationic dyes from water.

## Supplementary Information

Below is the link to the electronic supplementary material.

Supplementary Material 1

## Data Availability

No datasets were generated or analysed during the current study.
