# Supplementary material for: Sustainable synthesis of Ag/UiO-66 composites using recycled silver for efficient adsorption of methylene blue and crystal violet
Source: Sci Rep. 2026 Aug 11;16:24904. doi: 10.1038/s41598-026-65189-x (PMC13458786; doi:10.1038/s41598-026-65189-x)
Supplement: Supplementary file 1 [file 41598_2026_65189_MOESM1_ESM.docx]

**Supporting Information**

**Sustainable synthesis of Ag/UiO**‑**66 composites using recycled silver for efficient adsorption of methylene blue and crystal violet**

Hazem Ibrahem*, Hassanien Gomaa*, Ahmed A. Gahlan, Adham M.Nagiub*

Department of Chemistry, Faculty of Science, Al-Azhar University, Assiut, 71524, Egypt.

* Corresponding authors: (Hazem Ibrahem: [Ghazx1611@gmail.com](mailto:Ghazx1611@gmail.com)); Hassanien Gomaa ([h.gomaa@azhar.edu.eg](mailto:h.gomaa@azhar.edu.eg)); Adham M. Nagiub ([adham.nagiub@azhar.edu.eg](mailto:adham.nagiub@azhar.edu.eg))

**Fig. S1** Real images of MCCB waste and Silver-based contact alloys.

**Fig. S2** XPS survey spectrum of the Ag/UiO-66 composite, showing the O 1s, Zr 3p, Ag 3d, C 1s, and Zr 3d core-level peaks.

**Fig. S3** BJH pore-size distributions of (a) UiO-66 and (b) Ag/UiO-66 samples.

**Fig. S4** FTIR spectra of Ag/UiO-66 before adsorption and after adsorption of MB (MB@Ag/UiO-66) and CV (CV@Ag/UiO-66).

**Fig. S5** XPS survey spectra of Ag/UiO-66 before adsorption and after adsorption of MB (MB@Ag/UiO-66) and CV (CV@Ag/UiO-66).

**Fig. S6** PXRD patterns of Ag/UiO-66 before use (as-synthesized) and after the 5^th^ adsorption–desorption cycle.
